# DNMT3b protects centromere integrity by restricting R-loop-mediated DNA damage

**DOI:** 10.1038/s41419-022-04989-1

**Published:** 2022-06-11

**Authors:** Hsueh-Tzu Shih, Wei-Yi Chen, Hsin-Yen Wang, Tung Chao, Hsien-Da Huang, Chih-Hung Chou, Zee-Fen Chang

**Affiliations:** 1 Institute of Molecular Medicine, National Taiwan University, Taipei, 10051 Taiwan; 2 Center of Precision Medicine, College of Medicine, National Taiwan University, Taipei, 10051 Taiwan; 3 Institute of Biochemistry and Molecular Biology, National Yang Ming Chiao Tung University, Taipei, 11221 Taiwan; 4 Cancer Progression Research Center, National Yang Ming Chiao Tung University, Taipei, 11221 Taiwan; 5 Warshel Institute for Computational Biology, The Chinese University of Hong Kong, Longgang District, 518172 Shenzhen, China; 6 School of Life and Health Sciences, The Chinese University of Hong Kong, Longgang District, 518172 Shenzhen, China; 7 School of Medicine, The Chinese University of Hong Kong, Longgang District, 518172 Shenzhen, China; 8 Department of Biological Science and Technology, National Yang Ming Chiao Tung University, Hsinchu, 30010 Taiwan; 9 Center for Intelligent Drug Systems and Smart Bio-devices (IDS2B), National Yang Ming Chiao Tung University, Hsinchu, 30010 Taiwan

**Keywords:** Double-strand DNA breaks, DNA methylation, Primary immunodeficiency disorders

## Abstract

This study used DNA methyltransferase 3b (DNMT3b) knockout cells and the functional loss of DNMT3b mutation in immunodeficiency-centromeric instability-facial anomalies syndrome (ICF) cells to understand how DNMT3b dysfunction causes genome instability. We demonstrated that R-loops contribute to DNA damages in DNMT3b knockout and ICF cells. More prominent DNA damage signal in DNMT3b knockout cells was due to the loss of DNMT3b expression and the acquirement of p53 mutation. Genome-wide ChIP-sequencing mapped DNA damage sites at satellite repetitive DNA sequences including (peri-)centromere regions. However, the steady-state levels of (peri-)centromeric R-loops were reduced in DNMT3b knockout and ICF cells. Our analysis indicates that XPG and XPF endonucleases-mediated cleavages remove (peri-)centromeric R-loops to generate DNA beaks, causing chromosome instability. DNMT3b dysfunctions clearly increase R-loops susceptibility to the cleavage process. Finally, we showed that DNA double-strand breaks (DSBs) in centromere are probably repaired by error-prone end-joining pathway in ICF cells. Thus, DNMT3 dysfunctions undermine the integrity of centromere by R-loop-mediated DNA damages and repair.

## Introduction

DNA methyltransferase DNMT3b carries out de novo DNA methylation and is essential for mammalian development [1, 2]. The murine embryonic fibroblasts derived from *DNMT3b* knockout embryo display DNA damage and chromosome instability [1, 3], suggesting the critical function of DNMT3b in genome stability. Loss-of-function mutation in *DNMT3b* is specifically found in a rare human genetic disorder, immunodeficiency-centromeric instability-facial anomalies (type-1 ICF) syndrome [4–6]. It has been shown that DNMT3b is recruited to GC-rich (peri-)centromere regions by interacting with centromere protein CENP-C for maintaining chromosome stability [7]. In accordance, the major genome regions affected by the loss of DNMT3b function in ICF are non-coding repetitive elements surrounding centromeres, where GC regions are hypo-methylated [8], coinciding with centromeric DNA breaks observed in ICF cells [9, 10]. However, it is still an open question how DNMT3b dysfunction increases DNA damage and centromere instability.

R-loop is a three-stranded nucleic acid structure consisting of an RNA-template DNA hybrid and a non-template single-stranded DNA [11]. In mitosis, centromeric domain remains transcriptionally active [12]. It has been reported that centromeric R-loop has a function in mediating ATR binding for checkpoint control in mitosis [13], suggesting the beneficial role of R-loops in a cell. However, R-loops are also considered the source of DNA damage [14, 15]. R-loops can be resolved by helicases, translocase, or removed by RNase H [16–18]. Without a resolution, R-loops lead to DSBs through replication fork collapse [14] or DNA cleavage by endonucleases XPG and XPF [19, 20]. In addition, it has been shown that chromatin modifications regulate R-loop-induced genome instability [21–24]. Since DNMT3b is an epigenetic factor that regulates transcription, we asked the question of whether the levels and processing of R-loops are involved in DNMT3b dysfunction-mediated chromosome instability.

In this study, we addressed the question by using human colon cancer HCT116-BKO cells in which two alleles of *DNMT3b* disrupted by homologous recombination and ICF cells that carry loss-of-function mutations in *DNMT3b*. Our analysis demonstrated that R-loops contribute to prominent DNA damage signals observed in both *DNMT3b* knockout in HCT116 (BKO) and the loss-of-function mutation of DNMT3b in ICF lymphocytes. DNA damage sites in BKO cells were mapped to repetitive satellite sequences and rDNA genes. We focused on understanding the role of DNMT3b in centromere instability. In BKO and ICF cells, (peri-)centromeric R-loops are cleaved and removed by endonucleases XPG and XPF. Depletion of XPG and XPF elevated R-loops while reduced γH2AX associated with (peri)-centromeric DNA sequences in BKO and ICF cells. Finally, we examined the choice of DNA double-strand break (DSB) repair pathway in centromeric breaks in ICF cells. Our data indicate that error-free homologous recombination (HR) of DSB repair at centromere sites is not activated in ICF cells. Instead, the non-homologous end-join process (NHEJ), which might cause error-prone repair [25]. Here, we proposed that DNMT3b dysfunction promotes XPG/XPF-mediated DNA breaks at (peri-)centromeric R-loops sites, where the repair of DSBs via NHEJ increases centromeric shortening and fusion that undermine centromere stability in ICF cells.

## Results

### DNMT3b dysfunctions increase R-loops-dependent DNA damage

**Fig. 1:**
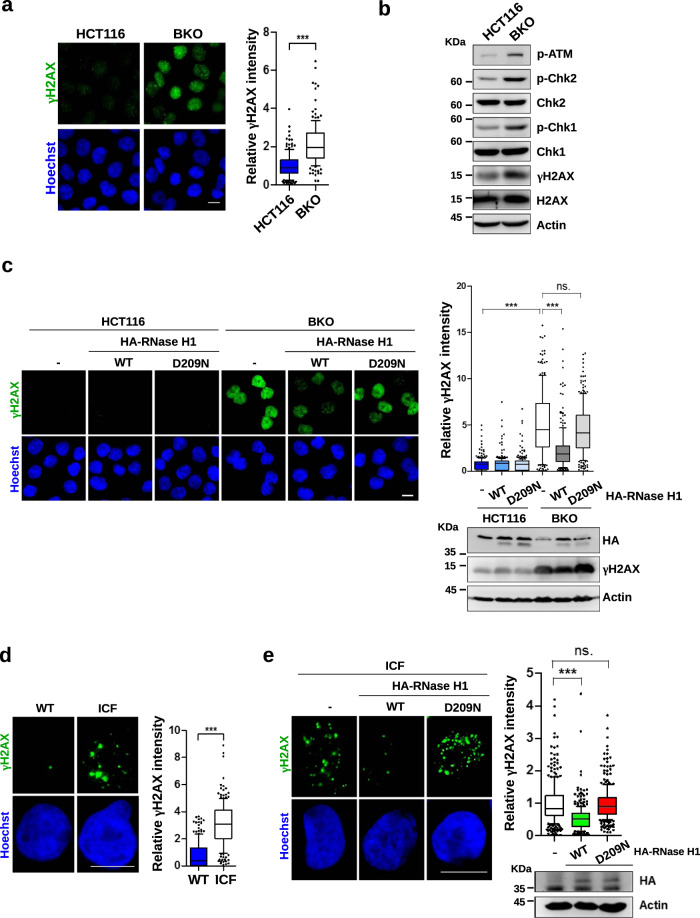
R-loops are the sources of DNA damage in BKO and ICF cells. **a**, **b** HCT116 and BKO cells were fixed for **a** γH2AX IF staining (scale bar, 10 μm). The relative intensity of γH2AX in cells (*n* > 150) from three independent experiments is expressed, ***P < 0.001 by the Mann–Whitney test. **b** Western blot analysis of DNA damage response markers. **c** γH2AX IF staining of HCT116 and BKO cells that were infected with retrovirus of empty HA-vector, HA-RNaseH1-WT, and HA-RNaseH1-D209N (scale bar, 10 μm, *left*). Relative fluorescent intensity of γH2AX in cells (*n* > 150) from three independent experiments (*upper right)*. Western blot of the expression of HA-RNase H1 (*bottom right)*. **d** The comparison of DNA damage signal in wild-type versus ICF LCL. Cells were fixed for IF staining by γH2AX antibody (scale bar, 10 μm). The relative intensity of γH2AX in cells (*n* = 100) from three independent experiments. **e** ICF LCLs were infected with retrovirus of empty HA-vector, HA-RNaseH1-WT, and HA-RNaseH1-D209N for γH2AX IF staining (scale bar, 10 μm, *Left*). Relative fluorescent intensity of γH2AX in cells (*n* = 100) from three independent experiments (*upper right)*. Western blot analysis of HA-RNaseH1-WT and HA-RNaseH1-D209N (*bottom right*).

To assess the importance of DNMT3b in genome instability, the levels of DSBs in HCT116 and BKO cells were compared by γH2AX IF staining. Data showed that the γH2AX signal was very prominent all over the nuclei in BKO cells (Fig. 1a), so as other DNA damage response signals, including phospho-ATM, -Chk2, and -Chk1 (Fig. 1b). Enforced expression of HA-RNase H1 significantly reduced the γH2AX signal in BKO cells (Fig. 1c). In contrast, the expression of HA-RNase H-D209N, a catalytic-dead mutant, had no effect (Fig. 1c). Since R-loops are the results of transcription, we then treated BKO cells with α-Amanitin (20 μg/ml) or cordycepin (50 μM) for 6 h and found that γH2AX intensity was clearly reduced (Fig. S1). These results suggest that R-loops contribute to the increase of DNA damage in BKO cells. A previous report has shown that transcription-coupled nucleotide excision repair (TC-NER)-mediated endonuclease cleavage of unprocessed R-loops promotes genome instability [19, 20]. We then depleted Cockayne Syndrome B (CSB) protein, a critical TC-NER factor in BKO cells. The overall γH2AX intensity was reduced in BKO cells after the knockdown of CSB, suggesting the involvement of NER in making DSBs in these cells (Fig. S2). It has been suggested that in the S phase, DNA gaps generated by TC-NER processing cause DNA replication fork collapses and DSBs, which activate γH2AX [26]. The flow cytometric analysis, showed that BKO cells contained more S phase cells (Fig. S3a). The γH2AX signal associated with the EdU incorporation-positive cells indicated by Click-iT conjugation was obviously higher in BKO cells (Fig. S3b). The DNA fiber analysis of replicating DNA further revealed that the lengths of replication tracks were much shorter in BKO than those in HCT116 cells (Fig. S3c). Altogether, these data suggest that the replication stress in the S phase is associated with DNA damage in BKO cells.

Next, we tested whether R-loops also cause DNA damage in ICF cells. To this end, we first compared γH2AX IF staining in EBV-immortalized lymphoblastoid cell lines (LCLs) from wild-type and a type-1 ICF patient carrying *DNMT3b* mutation (Fig. 1d). The results showed that the levels of γH2AX foci were higher in ICF than those in wild-type LCLs. We then used retroviral infection to express RNase H1 in ICF LCLs. The amounts of γH2AX foci were clearly decreased by wild-type but not catalytic-dead RNase H1 in ICF cells (Fig. 1e). In conclusion, either deficiency or functional loss of DNMT3b increases R-loops-dependent DNA damage.

**Fig. 2:**
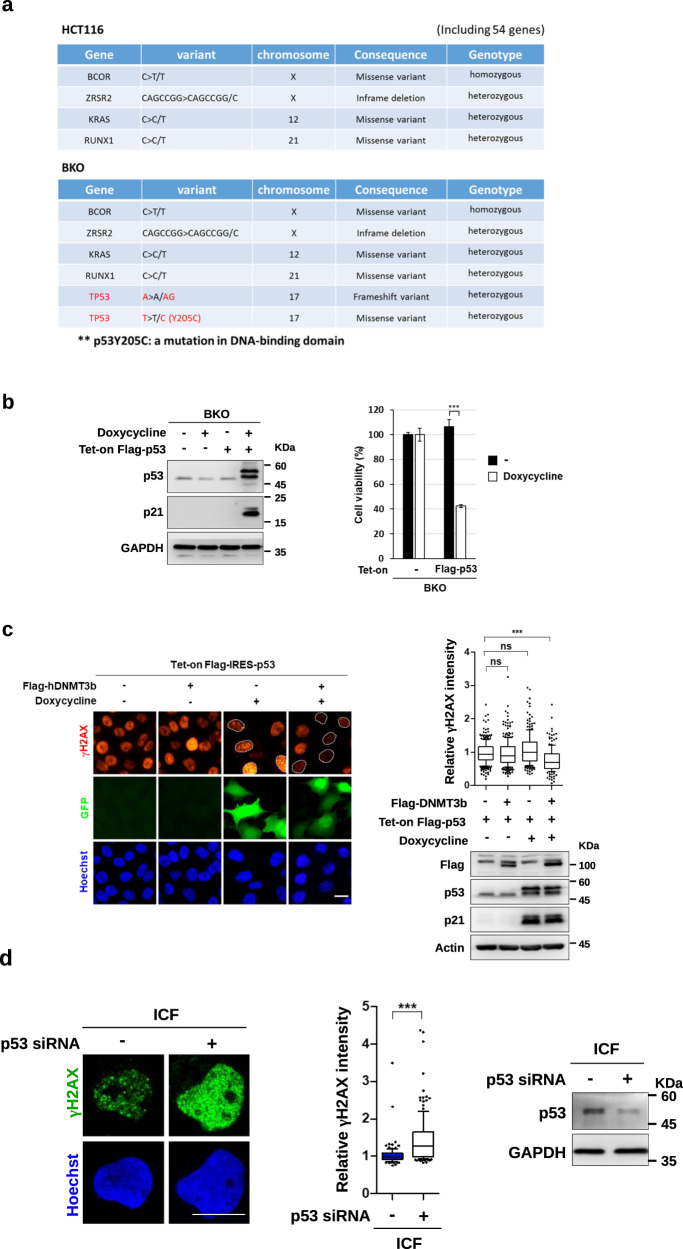
The loss of DNMT3b and the acquired p53 mutation cause prominent DNA damage in HCT116 cells. **a** NGS targeting sequencing of 54 genes in HCT116 and BKO cells. HCT116 and BKO shared common mutations in four genes. Additional mutations at p53 (*TP53*) were found in BKO cells. **b** BKO cells were infected with Tet-on Flag-p53-IRES-GFP virus followed by treatment with or without 2 μM doxycycline for 3 days for western blot (*left*) and viability assay (*right*, means ± SEM, *n* = 3, *** indicated *P* < 0.001 by two-tailed unpaired Student’s *t*-test. ns. *P* > 0.05). **c** BKO cells stably expressing Flag-DNMT3b were selected and infected with Flag-p53-IRES-GFP virus. Afterward, cells were treated with or without 1 μM doxycycline for 48 h and fixed for γH2AX IF staining (scale bar, 10 μm, *left*). Relative intensity of γH2AX in cells (*n* > 80), *** *P* < 0.001 by Mann–Whitney test (*upper right)*. Western blots of Flag-DNMT3b, p53, p21, and Actin (*bottom right)*. **d** ICF LCLs were transfection with 2 μg control siRNA or p53 siRNA using Amaxa® Cell Line Nucleofector® Kit V. After post-transfection at 48 h, cells were fixed for γH2AX IF staining (scale bar, 10 μm, *left*). Fluorescent intensity of γH2AX in cells (*n* > 100) was quantitated by Image J and relative intensity is expressed, ***P < 0.001 by the Mann–Whitney test (*middle)*. Western blots of p53 and GAPDH (*right*).

It is worthy of noting that the intensity of γH2AX was much more prominent in BKO than in ICF cells. Despite of high DNA damage signal, BKO cells still grew well in the culture. The sequencing of a panel of genes associated with cancer indicated *p53* mutation in BKO cells (Fig. 2a). Since BKO cells were generated by flox-mediated knockout from HCT116, which is a p53 proficient line, it is likely that R-loop-mediated DNA damages drive the acquirement of *p53* mutation in BKO cells. We found that tet-on induced expression of p53 by doxycycline treatment caused BKO cell growth arrest and cell death (Fig. 2b). Thus, the selection of p53 mutation allows BKO cell survival and proliferation. In addition, re-expression of both DNMT3b and p53 reduced the DNA damage signal in BKO cells (Fig. 2c). Very likely, the loss of functional p53 and DNMT3b expression together contribute to sustaining a prominent level of DNA damage in BKO cells. As for ICF cells, the intensity of the DNA damage signal was less, probably because p53 was still functional and *DNMT3b* gene was not truncated. Knockdown of p53 in ICF cells was performed to test the contribution of functional p53 to the level of endogenous DNA damage. The results showed that p53 knockdown clearly increased γH2AX foci in ICF cells (Fig. 2d). Thus, p53 plays a role in determining the extent of DNA damage induced by the loss of DNMT3b function.

### R-loops-dependent DNA damages at (peri-)centromere

**Fig. 3:**
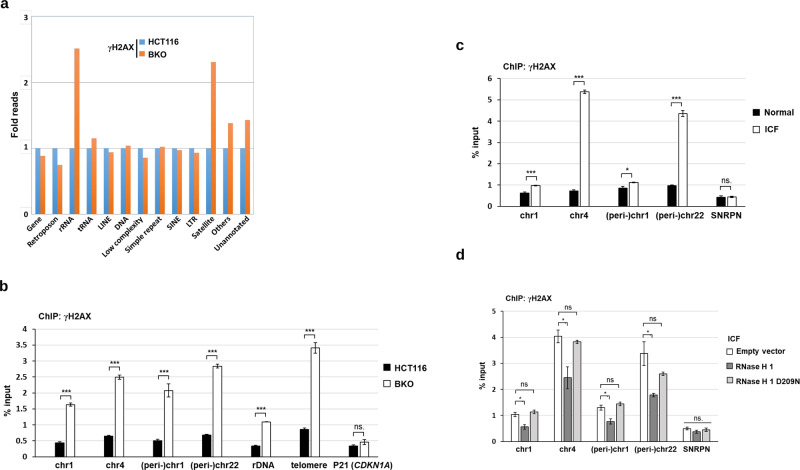
R-loops-dependent DNA damages at repetitive sequences. **a** Genome-wide sequencing of DNA damage sites in HCT116 and BKO cells. ChIP by γH2AX antibody was used for sequencing analysis. The plot shows the relative ChIP-seq reads of γH2AX at indicated genomic regions in BKO versus control HCT116 cells. Data were from one experiment. **b** HCT116 and BKO cells were used for γH2AX-ChIP-qPCR analysis. Data are shown as the percentage of input DNA in γH2AX antibody at the sequences of (peri-)centromere, rDNA, telomere, and control p21 (*CDKN1A*) regions (mean ± SEM, *n* = 3, ***P < 0.001, ns: no significant by two-tailed unpaired Student’s *t*-test). **c** γH2AX-ChIP-qPCR in wild-type and ICF LCL cells. γH2AX-ChIP-qPCR was analyzed at the sequences of (peri-)centromere of chromosome and intergenic region downstream of *SNRPN*. Data are expressed as percentage of input DNA (mean ± SEM, *n* = 3, *, *** *P* < 0.05, 0.001, ns: no significant by two-tailed unpaired Student’s *t*-test). **d** γH2AX-ChIP-qPCR analysis in ICF LCLs after infection with retrovirus of empty HA-vector, HA-RNaseH1-WT, or HA-RNaseH1-D209N. Data are expressed as percentage of input DNA (mean ± SEM, *n* = 3, *, ***P < 0.05, 0.001, ns: no significant by two-tailed unpaired Student’s *t*-test).

To map DNA damage sites in BKO cells, we performed a ChIP-sequencing analysis using the γH2AX antibody. This genome-wide search showed higher levels of γH2AX associated with satellite regions of telomere, centromere, peri*-*centromere, and rDNA in BKO cells (Fig. 3a). These data are correlated with transcription abnormalities previously observed at (peri-)centromere in *DNMT3b*-depleted HCT116 cells [7], telomeres in ICF cells [27], and rDNA in *DNMT1/DNMT3b*-disrupted HCT116 cells [28]. We further validated ChIP-seq data by ChIP-qPCR of these repetitive sequences. Several high complexity regions of (peri-)centromere were chosen for primer design. The results confirmed more DSBs in satellite regions of rDNA, telomere, and (peri*-*)centromere in BKO cells (Fig. 3b).

We also performed a ChIP-qPCR analysis to know whether ICF cells have more γH2AX binding to (peri-)centromere. The ChIP data revealed higher levels of γH2AX associated with centromere core sequences of chromosomes 1 and 4 and peri-centromeric sequences of chromosomes 1 and 22 in ICF LCL than in normal LCL (Fig. 3c). *SNRPN* locus has been commonly used as a negative control of R-loop hybrid [13, 29]. No difference in γH2AX ChIP at *SNRPN* was found in WT and ICF LCLs. To test the causal role of R-loops in (peri-)centromeric DSBs, we then expressed RNase H1 by retroviral infection in ICF cells. γH2AX-ChIP analysis showed that expression of wild-type of RNase H1 significantly reduced γH2AX association with (peri-)centromeric sequences in ICF cells, while catalytic-dead RNase H1 had little effect (Fig. 3d). These data suggest that R-loop formation increases γH2AX associated with (peri-)centromeric sequences.

### XPG- and XPF-mediated cleavages remove (peri-)centromeric R-loops and generate DNA damages in DNMT3b defective cells

**Fig. 4:**
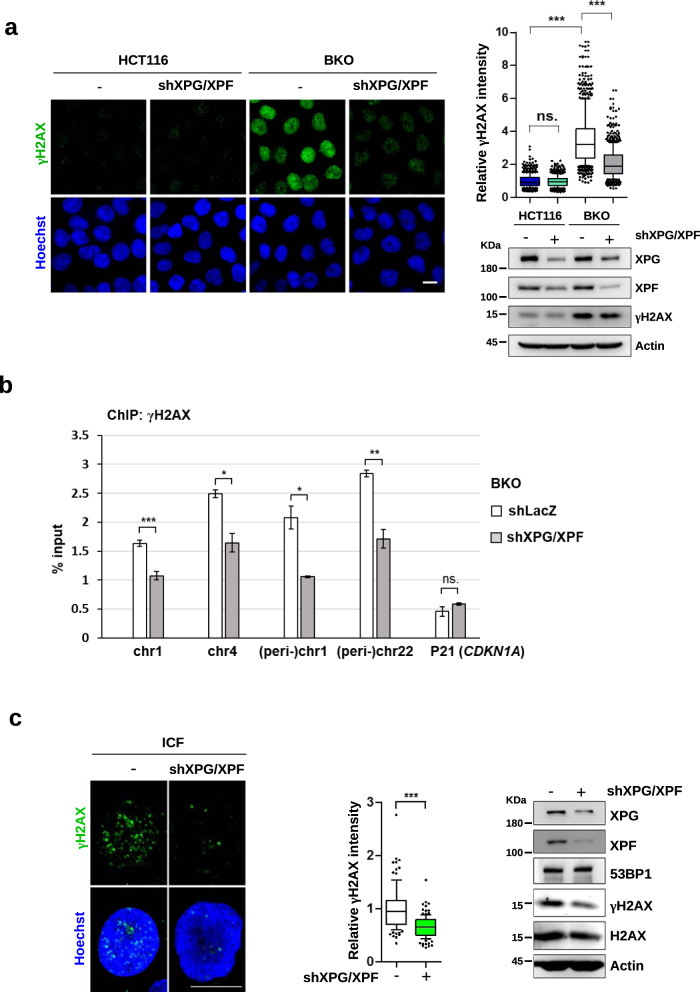

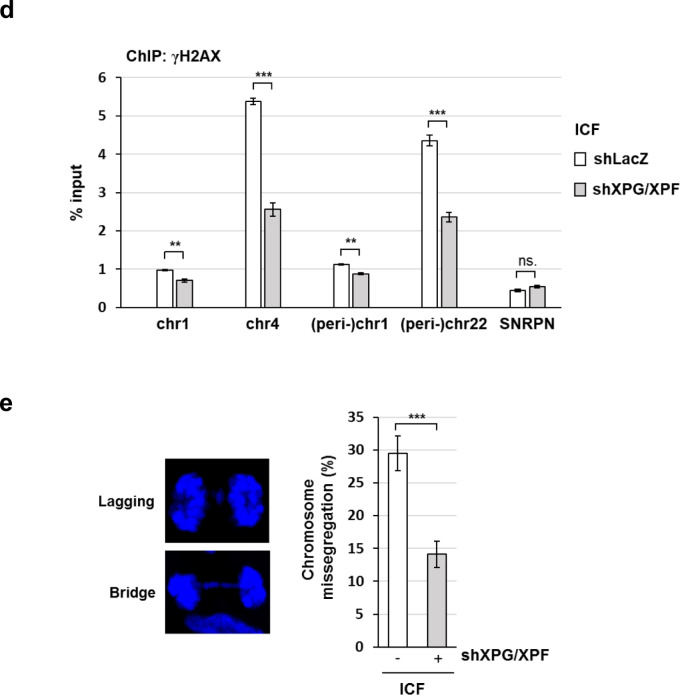
XPG and XPF cause centromeric DNA breaks in BKO and ICF cells. **a**, **b** HCT116 and BKO cells were infected with shXPG and shXPF lentivirus subsequently. After recovery and selection, cells were analyzed by γH2AX IF staining and γH2AX-ChIP-qPCR analysis. **a** IF staining images (scale bar, 10 μm, *left*). Fluorescent intensity was quantitated (*n* > 150) from three independent experiments, ***P < 0.001 by the Mann–Whitney test (*upper right*). Western blots of XPG, XPF, γH2AX, H2AX, and Actin (*bottom right*). **b** Data of γH2AX-ChIP-qPCR are expressed as % input (mean ± SEM, *n* = 3, *, ***P < 0.05, 0.001, ns: no significant by two-tailed unpaired Student’s *t*-test). **c**, **d** ICF LCLs were infected by LacZ and shXPG and shXPF lentivirus. After selection, cells were analyzed by γH2AX IF staining and γH2AX-ChIP-qPCR analysis. **c** IF staining images of γH2AX *(*Scale bar,10 μm, *left)*. Fluorescence Intensity was quantitated (*n* > 100) from three independent experiments and relative intensity is expressed, ***P < 0.001 by the Mann–Whitney test (*middle*). Western blots of XPG, XPF, γH2AX, H2AX, 53BP1, and Actin (*right*). **d** Data of γH2AX-ChIP-qPCR analysis are expressed as described in Fig. 3b (mean ± SEM, *n* = 3, *, **P < 0.05, 0.01, ns: no significant by two-tailed unpaired Student’s *t*-test). **e** Effect of XPG/XPF knockdown on chromatin segregation. ICF LCLs with and without XPG and XPF knockdown were arrested in mitosis by nocodazole treatment overnight. Cells were released from nocodazole arrest for 70 min and stained with Hoechst. Representative images of chromatin lagging and bridge errors are shown on the left. Percentages of cells with anaphase bridges and lagging chromosomes are shown in the histogram. Error bars are shown in means ± SEM, *n* = 3. *, *** indicate *P* < 0.05 and 0.001, respectively, by two-tailed unpaired Student’s *t*-test.

XPF and XPG are the endonucleases of TC-NER. We then depleted XPF and XPG, by which γH2AX IF staining in BKO cells was abolished (Fig. 4a). Consistently, ChIP-qPCR confirmed that γH2AX binding at (peri-)centromeric sites was also reduced by XPG and XPF knockdown (Fig. 4b). In ICF LCLs, depletion of XPG and XPF also abolished γH2AX foci (Fig. 4c). XPG/XPF knockdown did not affect the protein levels of 53BP1 and H2AX, excluding the possibility of their expression regulated by XPG/XPF. The comet assay also showed the reduction in the tail moment by XPG/XPF knockdown (Fig. S4). The γH2AX-ChIP-qPCR analysis also showed higher levels of γH2AX association with (peri-)centromeric sequences in ICF cells were also brought down by XPG/XPF depletion (Fig. 4d).

Centromere integrity is important in chromosome segregation. We then asked whether XPG/XPF knockdown can affect chromosome segregation in ICF cells. To this end, ICF cells were treated with nocodazole overnight. By washing out nocodazole, cells were allowed to have mitotic progression for chromosome segregation analysis. The amounts of lagging and bridge chromosome in mitosis were evaluated. The population of cells with lagging and bridge chromosomes in ICF cells was diminished by XPG/XPF knockdown (Fig. 4e). Thus, the loss of DNMT3b function promotes R-loop-mediated DNA damage via XPG/XPF cleavage, which contributes to chromosome instability.

### NER-mediated removal of (peri-)centromeric R-loops and Pol II stalling in DNMT3b knockout and ICF cells

**Fig. 5:**
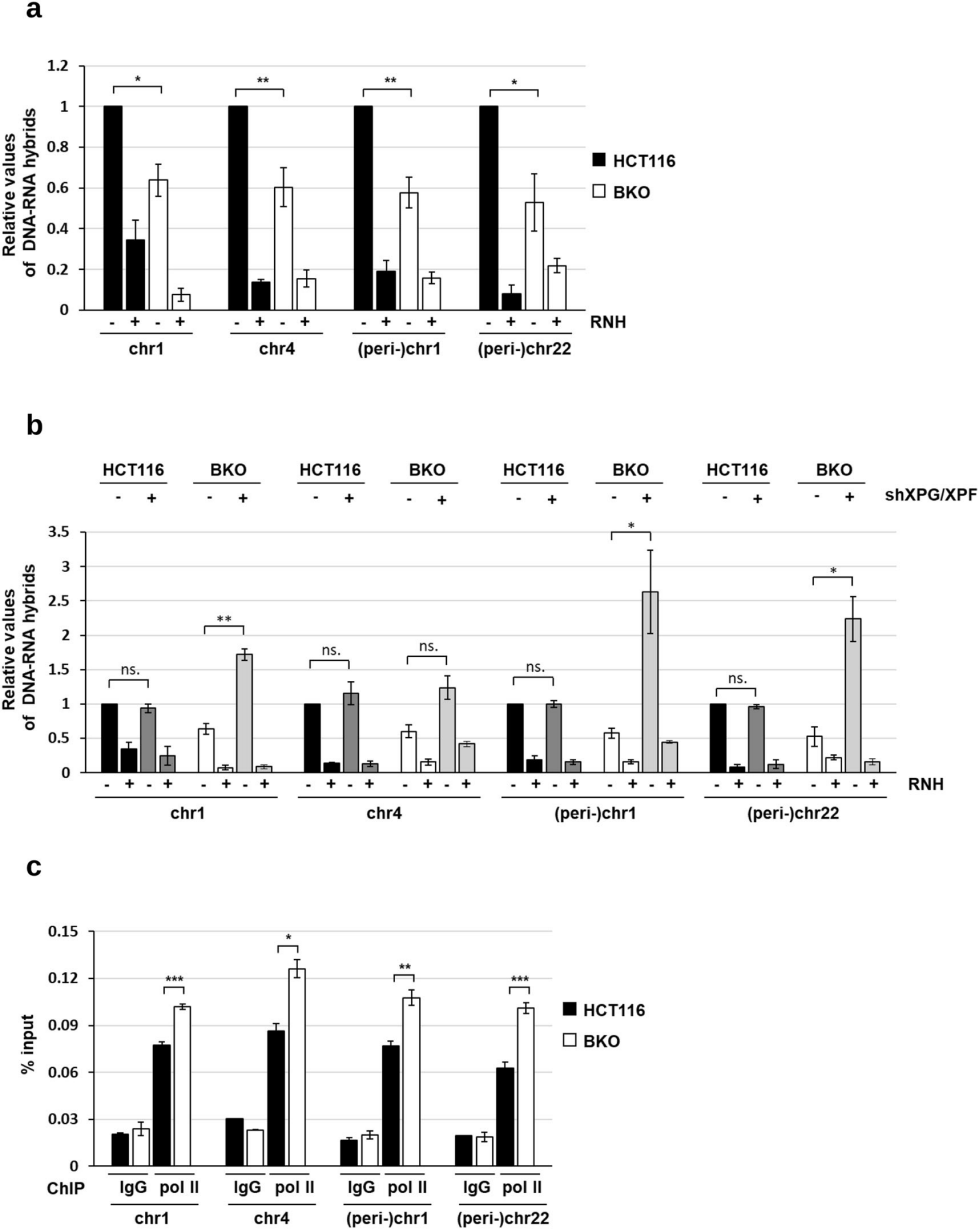
The regulation of (peri-)centromeric R-loops by XPG/XPF and transcription in BKO cells. **a**, **b** Levels of (peri-)centromeric R-loops by DRIP analysis. **a** HCT116 and BKO cells and **b** cells with or without XPG/XPF knockdown. All DNA samples were untreated (-) or treated (+) with RNase H before immunoprecipitation by S.9.6 antibody. qPCR values of DNA-RNA hybrids at (peri-)centromere sequences of chromosomes are normalized by IgG control. Data are expressed relative to that in HCT116 cells and presented as mean ± SEM of three independent experiments (*, **P < 0.05, 0.01, ns: no significant by two-tailed unpaired Student’s *t*-test, RNH: RNase H). **c** HCT116 and BKO cells were used for Pol II-ChIP-qPCR analysis. Data are shown as the percentage of input DNA in Pol II antibody versus IgG control at (peri-)centromere sequences of chromosomes regions (mean ± SEM, *n* = 3, *, **, ***P < 0.05, 0.01 0.001, ns: no significant by two-tailed unpaired Student’s *t*-test).

Next, the levels of (peri-)centromeric R-loops were compared in HCT116 and BKO cells. We then performed DRIP analysis using DNA-RNA hybrid recognition antibody S9.6 followed by qPCR of (peri-)centromeric region. After nucleic acid extraction, all samples were immuno-precipitated by S9.6 antibody. Samples pretreated with RNase H were used as the control. DRIP readouts normalized by those with RNase H pretreatment indicate the level of R-loop. Unexpectedly, the analyses revealed that the steady-state levels of (peri-)centromeric R-loop in HCT116 was indeed higher than those in BKO cells (Fig. 5a). DRIP analysis showed that XPG/XPF depletion brought up the level of centromeric R-loop in BKO but not WT HCT116 cells (Fig. 5b). Thus, in BKO cells, (peri-)centromeric R-loops are removed by NER-mediated cleavage to generate DNA damages. These results explained why the steady-state levels of (peri-)centromeric R-loops was lower in BKO cells.

Considering the recruitment of NER factors involves the stalling of RNA polymerase II (pol II), we then examined the association of pol II with (peri-)centromere sequence. The results showed that BKO cells had higher amount of pol II binding to (peri-)centromeric sequence (Fig. 5c). Taken together, these results suggest that the loss of DNMT3b expression causes pol II stalling in (peri-)centromeric regions, which not only generates R-loops but also increases NER recruitment to give DNA breaks.

**Fig. 6:**
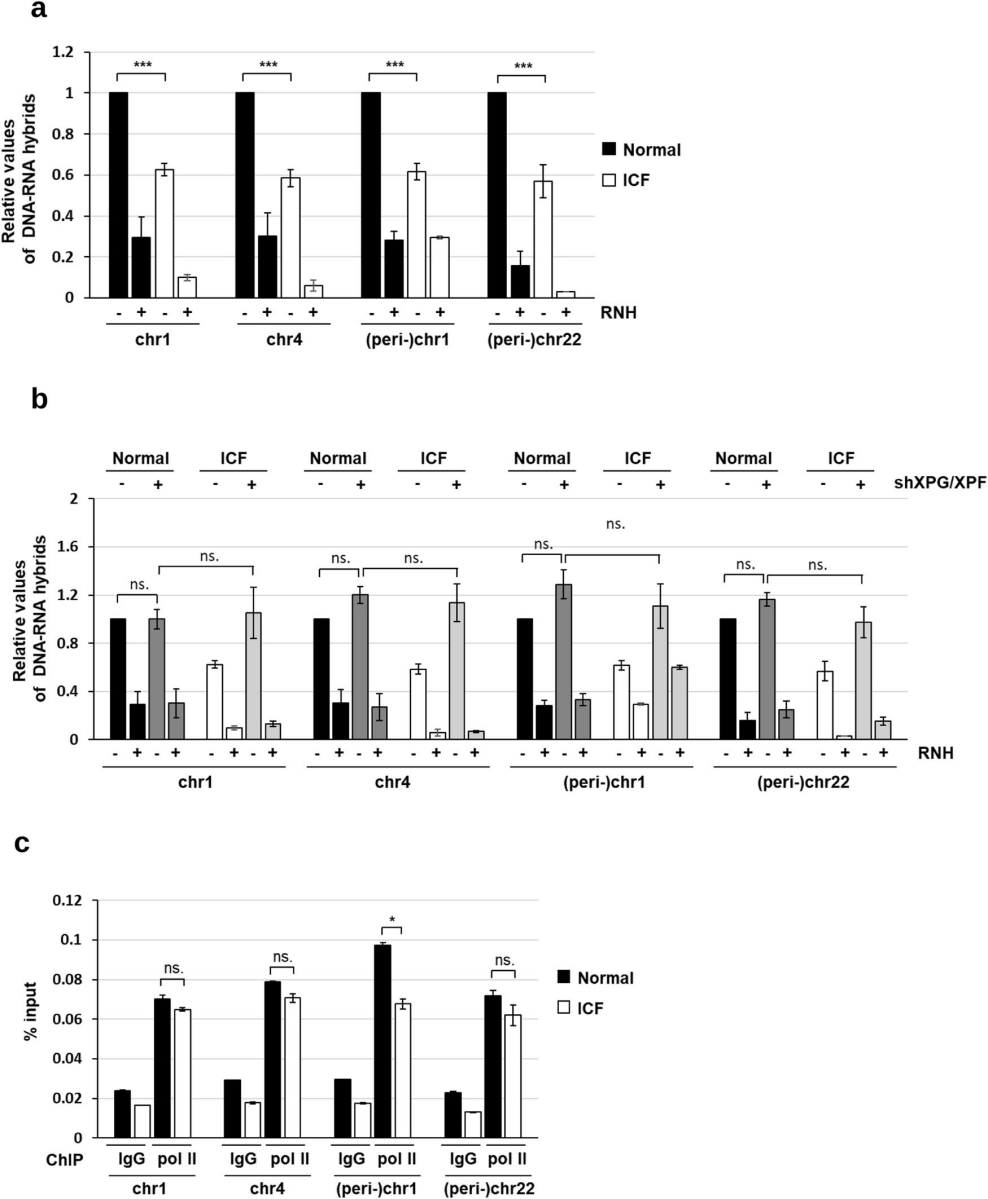
The functional mutation of DNMT3b in ICF increases XPG/XPF accessibility to (peri-)centromeric R-loops. **a**, **b** Levels of (peri-)centromeric R-loops by DRIP analysis. **a** Wild-type and ICF LCL cells. **b** Cells with or without XPG/XPF knockdown. All DNA samples were untreated (–) or treated (+) with RNase H followed by S.9.6 antibody immunoprecipitation for qPCR analysis at (peri-)centromere sequences of chromosomes. qPCR values of DNA-RNA hybrids are normalized by IgG control. Data are expressed relative to that in wild type cells and presented as mean ± SEM of three independent experiments (*, **P < 0.05, 0.01, ns: no significant by two-tailed unpaired Student’s *t*-test, RNH: RNase H). **c** Pol II-ChIP-qPCR analysis in wild-type and ICF LCLs. Data are shown as % of input DNA in Pol II antibody versus IgG control at (peri-)centromere sequences of chromosomes regions (mean ± SEM, *n* = 3, *P < 0.05, ns: no significant by two-tailed unpaired Student’s *t*-test).

Like WT and BKO HCT116 cells, the levels of (peri-)centromeric R-loops were higher in normal than those in ICF LCL cells (Fig. 6a). Of note, the DRIP assay for evaluating the level of DNA-RNA hybrid at the (peri-)centromeric sequences was confirmed by the inverse effect by ectopic expression of WT and catalytic-dead RNase H1 in ICF cells (Fig. S5). The level of DNA-RNA hybrid at the (peri-)centromeric sequences was also elevated by XPG/XPF knockdown in ICF but not normal cells (Fig. 6b). However, it was noted that the levels of (peri-)centromeric DNA-RNA hybrid in normal and ICF LCLs after XPG/XPF knockdown were similar. In addition, pol II ChIP analysis showed that no difference in pol II association in these sequences in normal and ICF cells (Fig. 6c). These data suggest that DNMT3b functional defect in ICF cells probably does not increase R-loop formation but only causes (peri-)centromeric R-loops more susceptible to NER-mediated cleavage.

### The lack of HR repair in centromere

**Fig. 7:**
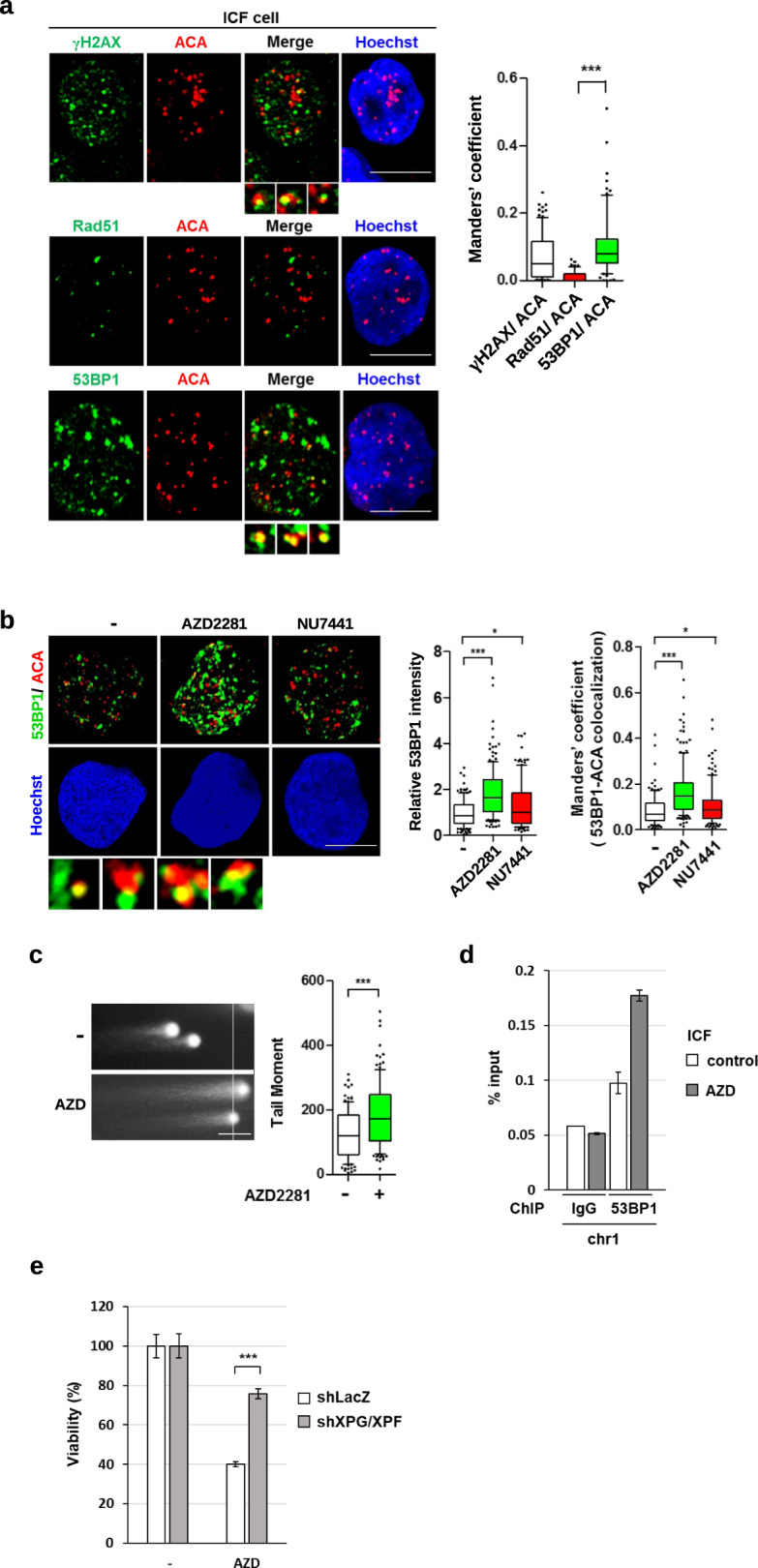
PARP1-mediated EJ repair in ICF cells. **a** ICF cells were fixed for γH2AX/ACA, Rad51/ACA, and 53BP1/ACA IF co-staining. Representative confocal co-localized signals (scale bar, 10 μm). Manders’ coefficient of co-localized γH2AX/ACA, Rad51/ACA, and 53BP1/ACA was acquired by confocal imaging as described in the methods. Examples of co-localized signals of γH2AX/ACA and 53BP1/ACA are shown under the Merged image. Co-localized signals are quantitated (*n* > 100) and expressed in relative values of Manders’ coefficient, ***P < 0.001 by the Mann–Whitney test. **b** ICF LCLs were treated with NU7441 (5 μM) or AZD2281 (5 μM) for 6 h and fixed for 53BP1/ACA IF co-staining. Representative images are shown (scale bar, 10 μm). The fluorescent intensity of 53BP1 was quantitated and relative intensity is expressed (*n* > 100), *, ***P < 0.001 by the Mann–Whitney test. Manders’ coefficient of co-localized 53BP1/ACA was quantified and shown. Examples of co-localized signals of 53BP1/ACA are shown under the images. Quantitation data of co-localized signal are expressed (*n* > 150), *, ***P < 0.05, 0.001 by the Mann–Whitney test. **c** and **d** ICF LCLs were treated with AZD2281 (5 μM) for 6 h for Comet and 53BP1-ChIP-qPCR analysis. **c** Comet tail moments in cells (*n* = 300) were analyzed by CometScore (***P < 0.001 by Mann–Whitney test). **d** Data of 53BP1-ChIP-qPCR are shown as the percentage of input DNA at the centromere sequence of chromosome 1 from two independent experiments. **e** Effect of XPG/XPF knockdown on AZD2281 sensitivity by viability assay. ICF LCLs with or without shXPG/XPF knockdown were treated with AZD2281 (5 μM) for 72 h. The percentage of the cell viability from three independent experiments is shown, mean ± SEM, ***P < 0.001 by two-tailed unpaired Student’s *t*-test.

It has been reported that DNMT3b mutation in ICF leads to centromere shortening [9, 10]. Presumably, endogenous DSBs are constantly repaired to prevent genome deterioration. We suspected that the repair of DNA breaks might lead to centromere shortening. DSBs are repaired by homologous recombination (HR) or non-homologous end-join (NHEJ) repair pathways [30]. HRR pathway involves an extended BRCA1-dependent end resection and loading of Rad51 for strand invasion to give error-free repair [31]. 53BP1 recruitment to DSB sites antagonizes the HRR pathway and favors NHEJ repair [31–33]. By confocal microscopic analysis, γH2AX and 53BP1 were well co-localized with the IF signal of anti-centromere antibody (ACA) that marks centromere sites. In contrast, little Rad51 foci were co-localized with ACA signal in ICF cells (Fig. 7a), suggesting the lack of ongoing HRR at centromeric sites. We then tested NHEJ in the repair of DNA damage in ICF cells. Two NHEJ pathways, conventional NHEJ (c-NHEJ) and alternative-end joining (Alt-EJ), participate in the repair of two-ended and one-ended DSBs [34]. NU7441, an inhibitor of DNA-PK, specifically suppresses c-NHEJ. AZD2281, a PARP1 inhibitor, suppresses multiple repair pathways including alt-EJ and c-NHEJ. ICF cells were then treated with NU7441 and AZD2281 to distinguish the contribution of these two NHEJ pathways in repairing endogenous DSBs. AZD2881 treatment significantly increased overall 53BP1 foci. The amounts of these foci co-localized with ACA were also increased. NU7441 treatment gave a much lesser effect on 53BP1 foci and their co-localization with ACA (Fig. 7b). Comet assay also showed that AZD treatment increased the length of the tail moment in ICF cells, confirming the contribution of PARP1 activity in reducing DNA damage in ICF cells (Fig. 7c). We further performed ChIP analysis by 53BP1 antibody in ICF LCLs with and without AZD treatment. The result showed that inhibition of PARP1 significantly increased 53BP1 binding at the centromere site of chromosome 1 (Fig. 7d), suggesting that the centromere DNA breaks are constantly repaired in a PARP1-dependent manner. As such, AZD treatment decreased the viability of ICF cells, whereas XPG/XPF knockdown ICF cells were less susceptible to this treatment (Fig. 7e). Likely, inhibition of PARP1 accumulates DSBs in the centromere. This could also occur in the repetitive regions such as rDNA and telomere, leading to cell death in ICF cells. These data suggest that DNA lesions at centromeric sites might be frequently repaired by the PARP1-mediated DNA repair pathway. Given the lack of error-free HRR, our data imply that XPG/XPF-induced DSBs in centromere and the participation of NHEJ repair leads to centromere instability in ICF cells.

## Discussion

This study investigated DNA damage in DNMT3b deficient HCT116 cells and the loss of DNMT3b function in ICF cells. Despite prominent differences in the intensity of DNA damage signals in type-1 ICF and BKO cells, the signals in these cells were R-loops-dependent. It has been found that DNMT3b dysfunction mainly causes hypomethylation in repetitive sequences rather than promoters of coding genes [1, 35]. Consistently, we found that R-loop-mediated DNA damages are spreading over these repetitive sequences at the centromere, telomere, and rDNA regions. Since XPG/XPF knockdown was able to abolish DNA damage signal in either BKO or ICF cells, it is apparent that R-loop-mediated DNA breaks in these sequences are the results of DNA cleavages by these two DNA endonucleases in the NER process. Moreover, our data also point out that a prominent DNA damage signal observed in BKO cells involves the loss of DNMT3b together with the acquired p53 mutation.

DNA hypomethylation not only affects transcription but also histone modification and recruitment of chromatin factors [36]. Our findings further addressed the question of whether the induction of R-loop-mediated DNA damages by DNMT3b dysfunction is through generating too many R-loops or having more DNA cleavages by XPG/XPF. The steady-state levels of (peri-)centromeric R-loops are higher in HCT116 and normal LCLs as compared to those in BKO and ICF cells, respectively. After XPG/XPF depletion, the centromeric R-loops were significantly increased in BKO and ICF but not HCT116 and normal LCLs. Thus, it is the NER-mediated cleavage in BKO and ICF cells that decrease the levels of centromeric R-loops. Depletion of XPG and XPF caused the level of centromeric R-loop in BKO cells higher than in HCT116 cells. The amount of Pol II binding to these regions was also higher in BKO cells. Contrarily, in normal and ICF LCLs after XPG/XPF knockdown, the levels of centromeric R-loops were similar. Therefore, the loss-of-function DNMT3b mutation increases the removal of centromeric R-loop by the NER process, rather than having excessive amounts of R-loop formation. In addition, we did not find an increase of pol II binding in ICF cells. Accordingly, we proposed that DNA methylation by DNMT3b at (peri-)centromere R-loop sites mediates an as-yet-unknown process in restricting the recruitment of NER factors, thus preventing R-loops in centromere from XPG/XPF-mediated processing. ICF cells defective in DNMT3b function, therefore, have more NER-dependent DSBs in the centromere, leading to centromere instability.

This study also investigated the repair of these R-loops-mediated DSBs in centromere. We did not find Rad51 foci, an HHR marker, localized at centromere sites. In contrast, 53BP1 foci were localized at centromeric sites, which would prevent HRR but favor NHEJ in DNA repair. This is consistent with a previous study showing that the HR repair factor is excluded from the heterochromatin domain to prevent abnormal recombination of repetitive sequences unless the DSB site moves outside the domain [37]. Although conventional NHEJ repair can cause small deletion to threaten the integrity of the coding gene, this problem might not be severe enough to affect repetitive satellite DNA sequences that span over the mega-base range. PARP1 is critical for many DNA repair pathways including c-NHEJ and alt-EJ pathways, while DNA-PK is required for c-NHEJ but not alt-EJ repair [38–40]. This study showed that inhibition of DNA-PK had a rather moderate effect on the overall 53BP1 foci, whereas PARP1 inhibition markedly increased 53BP1 foci associated with centromere. Furthermore, the ChIP analysis showed 53BP1 binding at centromeric sites increased by PARP1 inhibition. This is consistent with a recent report showing that the cell viability of two DNMT3b knockout clones of HEK293 cells is not affected by DNA-PK inhibitor treatment, while the cell viability of one clone was affected by PARP1 inhibitor [41]. Altogether, we conclude that PARP1-dependent repair of DNA lesions is constantly activated in type-1 ICF cells. Given the lack of error-free HHR in the centromere, we hypothesize that the repair of the centromeric DSBs generated by XPG/XPF-mediated removal of R-loops by the end-joining process might lead to centromere shortening, metaphase quadriradials with mixed chromosomal arms as common features observed in ICF due to DNMT3b dysfunction [9, 10].

## Materials and methods

### Cell culture, transfection, and infection

HCT116 and BKO cells were kindly provided by Bert Vogelstein (Johns Hopkins University School of Medicine, MD, USA) and were maintained in McCoy’s 5A medium supplemented with 10% fetal bovine serum (FBS), 100 U/ml penicillin and 10 mg/ml streptomycin. BKO cells were generated by disrupting the alleles of *DNMT3b* by homologous recombination in HCT116 cells [42]. Type-1 ICF cells, pGM08714 (lymphoblastoid cell lines, LCLs) was obtained from Coriell Cell Repositories. In pGM80714 (ICF) cells, one allele of *DNMT3b* has a nucleoside mutation at G1807 (1807G > A), which gives a substitution at codon 603 (A603T), and another allele has a 9-bp insertion, which results in three amino acids insertion at codon 744 (744ins3). Both mutations occur within the catalytic domain of *DNMT3b*. Normal (WT) LCLs were kindly provided by Prof. Ching-Hwa Tsai (Graduate Institute of Microbiology, National Taiwan University). LCLs were maintained in RPMI supplemented with 15% FBS, 2 mM glutamine, 100 U/ml penicillin, and 10 mg/ml streptomycin. HEK293T and Platinum-A retroviral packaging (Cell Biolabs, CA, USA) cells were maintained in Dulbecco’s modified Eagle’s medium supplemented with 10% FBS, 100 U/ml penicillin and 10 mg/ml streptomycin. HEK293T cells were cotransfected with pCMVdeltaR8.91, pCMV VSVG, and targeted plasmids including PL-SIN-5TO-Flag-p53-IRES-GFP, *ERCC4* (XPF) shRNA (TRCN0000078587) and *ERCC5* (XPG) shRNA (TRCN0000358878) to produce lentivirus. For LCL cells, individual shRNA viral infection was performed by incubation of cells with viral supernatant followed by centrifugation at 800 x *g* for 1 h and recovery for 2 days. For double knockdown by shXPG and shXPF viral infection, cells were infected with shXPG virus and selected with puromycin (1 μg/ml), followed by subsequent shXPF viral infection. After recovery for 2 days, cells were incubated in the growth medium containing puromycin (1 μg/ml). For retrovectors infection, pMXs-3XHA-mouseRNaseH1, pMXs-3XHA-mouseRNaseH1-D209N, and pQFlag-hDNMT3b were transfected into Platinum-A cells to produce retrovirus. After transfection for 48 h, the supernatants containing the virus were collected for infection.

### Antibodies and reagents

Antibodies: anti-DNA-RNA hybrid S9.6 (Millipore, MABE1095), HA (Santa Cruz, sc-805), Flag (Sigma-Aldrich, F3165), β-tubulin (Sigma-Aldrich, T4026), β-Actin (Santa Cruz, sc-8432), GAPDH (GeneTex, GTX100118), Chk1 (Santa Cruz, sc-8408), phosphoS345-Chk1 (cell signaling, #2341), Chk2 (Millipore, 05-649), phosphoT68-Chk2 (cell signaling, #2661), phosphoS1981-ATM (Gene Tex, GTX61739), phospho S139-H2AX (Millipore, 05-636), phospho S139-H2AX (Abcam, ab2893), H2AX (Gene Tex, GTX127340), XPF (Santa Cruz, sc-398032), XPG (Proteintech, 11331-1-AP), 53BP1 (Millipore, MAB3802), H3S10p (Millipore, 06-570), p53 (Calbiochem, OP43), p21 (Santa Cruz, sc-817), and Pol II (N-20) (Santa Cruz, sc-899). Chemicals for cell treatment: Nocodazole (Sigma, M1404), NU7741 (Santa Cruz, sc-208107), and AZD2281 (Selleckchem, S1060).

### Plasmids

The human DNMT3b expression vector, pQFlag-DNMT3b, was constructed by insertion of PCR products of pcDNA/Mcy-DNMT3b, which was purchased from Addgene (plasmid #35522), at *EcoR*I (5′) and *BamH*I (3′) to pQFlag vector. pMXs-3XHA-mouseRNaseH1 expression vectors were constructed by insertion of PCR products amplified from mouse RNaseH1 cDNA at *EcoR*I (5′) and *BamH*I (3′). pMXs-3XHA-mouseRNaseH1-D209N expression vectors were generated by site-specific metastasis. Tet-on expression vectors of PL-SIN-5TO-Flag-p53-IRES-GFP were constructed by insertion of PCR products amplified from p53 cDNA at *EcoR*I (5′) and *BamH*I (3′).

### Immunofluorescence staining

Cells were fixed with 4% paraformaldehyde for 15 min at room temperature (RT), followed by blocking in 5% BSA/TBS for 1 h at RT and staining with primary antibody overnight at 4 °C. After incubation with a secondary antibody for 1 h at RT, slides were mounted in Fluoro-gel mounting oil (EMS, #17985-10) and were analyzed by an OLYMPUS BX53 and LSM 700 laser scanning confocal microscope (Carl Zeiss).

### Comet assay

Comet assay was performed by Electrophoresis Assay (Trevigen, Inc) according to the manufacturer’s protocol. DNA was stained with ethidium bromide and analyzed with Image J (v 1.47) for measuring tail length (TL). The comet tail (TM = %DNA in tail × TL/100) according to the manufacturer’s suggestion.

### Chromatin immunoprecipitation (ChIP)-sequencing

Chromatin immunoprecipitation using the anti-gamma H2A.X (Abcam, ab2893) was carried out according to a previously described protocol [43]. Libraries were constructed and bar-coded using TruSeq RS Cluster kit-HS (Illumina) and single-end sequencing (50 bp) was performed using an Illumina HiSeq-2500 sequencer at the Genomic Research Center of National Yang-Ming University (Taiwan) according to the manufacturer’s instruction. All reads were mapped to the human genome (hg38) using the bowtie2 alignment software [44]. The alignment results were used to call peaks by MACS [45]. The results of the peak signal were subject to the Integrative Genomics Viewer (IGV, Broad Institute) [46] for further comparison and analysis. We used UCSC hg38 and Rfam v11 to identify and annotate the ChIP-seq peak regions.

### DNA-RNA immunoprecipitation (DRIP) assay

DRIP was performed as described in [47]. Briefly, DNA was extracted carefully by Wizard® Genomic DNA Purification Kit (Promega, A1125), washed with 70% EtOH, and resuspended in TE buffer. The purified nucleic acids were sonicated in a buffer containing 10 mM Tris-HCl pH 8.5 and 300 mM NaCl to yield an average DNA fragment size of ∼300 bp. Sonicated DNA (6 μg) was added in the IP buffer (50 mM HEPES/KOH at pH 7.5; 0.14 M NaCl; 5 mM EDTA; 1% Triton X-100; 0.1% Na-Deoxycholate), followed by addition of S9.6 antibody (10 μg) for incubation overnight at 4 °C. For removal of R-loops as a negative control, 6 μg of sonicated DNA was pretreated with 12 μl of RNase H (5000 U/mL; NEB, M0297s) for incubation at 37 °C for 6 h prior to immunoprecipitation by S9.6 antibody. Protein G Sepharose beads that were pre-blocked with PBS containing 0.5% BSA, were added for 4 h to pull down immunocomplex. Bound beads were recovered and washed with 1 ml of low-salt buffer (50 mM HEPES/KOH, pH 7.5, 0.14 M NaCl, 5 mM EDTA pH 8, 1% Triton X-100, 0.1% Na-Deoxycholate), 1 ml of lysis buffer (50 mM HEPES/KOH pH 7.5, 0.5 M NaCl, 5 mM EDTA pH 8, 1% Triton X-100, 0.1% Na-Deoxycholate), 1 ml of washing buffer (10 mM Tris-HCl, pH 8, 0.25 M LiCl, 0.5% NP-40, 0.5% Na-Deoxycholate, 1 mM EDTA pH 8), and 1 ml of TE (100 mM Tris-HCl, pH 8, 10 mM EDTA, pH 8) at 4 °C. Precipitates were eluted in elution buffer (10 mM Tris pH 8, 1 mM EDTA, 1% SDS) in 100 μl for 15 min at 65 °C. DNA was purified with QIAquick® PCR purification Kit (QIAGEN, 28106).

### qPCR for ChIP and DRIP

qPCR was performed on a StepOne™ Real-Time PCR System (Applied Biosystems™ LS4376357) using the Fast SYBR-Green master mix (Applied Biosystems™). qPCR primers are as followed:

Chr1 centromere, forward TCATTCCCACAAACTGCGTTG and reverse TCCAACGAAGGCCACAAGA; Chr4 centromere forward GTGGGAACCACA GAACCACT and reverse TTTCATGCGCCACCTTTTGG; Chr1 peri-centromere, forward CATCGAATGGAAATGAAAGGAGTC and reverse ACCATTGGATGATTGCAGTCAA; Chr22 peri-centromere, forward GCCTGTGTGTGAGGACGTAA and reverse GCCAATCTACCAGCCCACAT; Chr10 telomere, forward GTCCGTCCGTGAAATTGCG and reverse GGTCCAAACGAGTCTCCGTC; rDNA, forward CGATGGTGGCGTTTTTGG and reverse CCGACTCGGAGCGAAAGATA; p21, forward CTGCCCAAGCTCTACCTTCCCA and reverse GGTCCACATGGTCTTCCTCTGC; *SNRPN* as a control, forward GCCAAATGAGTGAGGATGGT and reverse TCCTCTCTGCCTGACTCC AT.

### Chromosome segregation analysis

Cells were treated with 0.2 μg/ml nocodazole for 16 h. After replacing with fresh medium for 1 h, cells were fixed with 4% paraformaldehyde for 15 min at RT, followed by blocking in 5% BSA/TBS for 1 h at RT and staining with mouse antibody to β-tubulin (1:200) and rabbit antibody to phosphoSer10- H3 (1:500) overnight at 4 °C. Cells were incubated with secondary antibodies in the presence of Hoechst 33342 for 1 h at RT. Cells were mounted in Fluoro-gel mounting oil (EMS, #17985-10) for observation by OLYMPUS BX53 and confocal microscope (ZEISS, LSM700).

### Gene-sequencing panel

TruSight® Myeloid Sequencing Panel (Illumina, San Diego, CA, United States) includes 54 genes from myeloid somatic mutations and uses Next-generation sequencing (NGS) technology to identify somatic variants. The 15 full genes (exons only) include *BCOR, BCORL1, CDKN2A, CEBPA, CUX1, DNMT3A, ETV6/TEL, EZH2, KDM6A, IKZF1, PHF6, RAD21, RUNX1/AML1, STAG2*, and *ZRSR2*, and oncogenic hotspots of 39 genes include *ABL1, ASXL1, ATRX, BRAF, CALR, CBL, CBLB, CBLC, CSF3R, FBXW7, FLT3, GATA1, GATA2, GNAS, HRAS, IDH1, IDH2, JAK2, JAK3, KIT, KRAS, KMT2A/MLL, MPL, MYD88, NOTCH1, NPM1, NRAS, PDGFRA, PTEN, PTPN11, SETBP1, SF3B1, SMC1A, SMC3, SRSF2, TET2, TP53, U2AF1*, and *WT1*.

### Confocal microscope and image quantification

Co-localization of two proteins of IF staining was acquired by LSM 700 laser scanning confocal microscope (Carl Zeiss). The conditions of IF staining, laser power, and pinhole sizes by confocal microscopy were identical among groups. Pixel number, pixel intensity, and area were provided by built-in software in LSM 700. Manders’ coefficient was calculated for the co-localization of two proteins in the nuclear area. The co-localization coefficient values are ranged from 0 (segregation) to 1 (complete co-localization).

### Cell viability assay

Cells were plated at 1,000 cells per well in a 96-well plate. Cells were treated with NU7441 (5 μM) or AZD2281 (5 μM) for 72 h, after which 10 μl of the Cell Counting Kit-8 (CCK-8) solution was added into each well for an additional 2 h at 37 °C. The absorbance at 450 nm was measured using a Tecan Spark multimode microplate reader and the percentage of cell viability was calculated.

### siRNA transfection

siRNA transfection was performed as described previously [48]. *TP53* (p53) was knocked down using human TP53 siRNA Smart Pool (L-003329-00-0010, Dharmacon) and siGLORNAi control (Dharmacon). The siRNA transfections were processed using Amaxa® Cell Line Nucleofector® Kit V for TP53 knockdown in ICF cells according to the manufacturer’s protocol. After 48 h transfection, cells were harvested and analyzed.

### Statistics

Statistical analysis was performed using an unpaired two-tailed Student’s *t*-test and Mann–Whitney test. Differences were considered statistically significant when *P*-values < 0.05, 0.01, and 0.001 indicated by *, **, and ***, respectively. Error bars represent the SEM of at least three independent experiments.

## Supplementary information

Supplemental information

Supplemental material (original data-blots)

Reproducibility checklist

Original Data File

## Data Availability

Sequencing data have been deposited at the GEO under the accession number GSE142376 and linked to UCSC genome browser session of DNMT3B study. https://genome.ucsc.edu/s/chenwy/rH2AX_ChIP%2Dseq_HCT116
