# Supplementary material for: DNMT3b protects centromere integrity by restricting R-loop-mediated DNA damage
Source: Cell Death Dis. 2022 Jun 11;13(6):546. doi: 10.1038/s41419-022-04989-1 (PMC9187704; doi:10.1038/s41419-022-04989-1)
Supplement: Supplementary file 1 — Supplemental information [file 41419_2022_4989_MOESM1_ESM.pdf]

Supplemental information

DNMT3b Protects Centromere Integrity by Restricting R-loop-mediated DNA damage

Hsueh-Tzu Shih[1,2], Wei-Yi Chen[3,4], Hsin-Yen Wang[1], Tung Chao[1], Hsien-Da Huang[5,6,7], Chih-Hung Chou[8,9], Zee-Fen Chang[1,2*]

[1] Institute of Molecular Medicine, [2] Center of Precision Medicine, College of Medicine, National Taiwan University, Taipei 10051, Taiwan

[3] Institute of Biochemistry and Molecular Biology, [4]Cancer Progression Research Center, National Yang Ming Chiao Tung University, Taipei 11221, Taiwan

[5] Warshel Institute for Computational Biology, [6] School of Life and Health Sciences, [7] School of Medicine, The Chinese University of Hong Kong, Shenzhen, 518172, Longgang District, Shenzhen, China

[8] Department of Biological Science and Technology, [9] Center for Intelligent Drug Systems and Smart Bio-devices (IDS^{2}B), National Yang Ming Chiao Tung University, Hsinchu 30010, Taiwan

* To whom correspondence should be addressed. Email: zfchang@ntu.edu.tw

Supplemental materials and methods

Antibodies and reagents

CSB (Santa Cruz, sc-166042). Other antibodies have been mentioned in main context. Chemicals for cell treatment: α-amanitin (SIGMA, A2263), and cordycepin (SIGAMA, C3394).

Flow cytometry

Cells were harvested and suspended in chilled 75% ethanol (4 °C). After incubation at 4 °C for 24 h, cells were washed with PBS and resuspended in PBS with propidium iodide (PI; 100 μg/ml) for 30 min at room temperature. The cell cycle progression was measured and analyzed using a Cell Quest software from BD FACSCalibur flow cytometer.

High-Content image analysis

To measure DNA damage in S phase cells, cells were pulse-labeled with 10 μM EdU for 30 min and processed with the Click-iT™ EdU Alexa Fluor™ 488 dye Imaging Kit according to manufacturer's protocol. For immunostaining, cells were fixed with 4% paraformaldehyde for 15 min at room temperature (RT), followed by blocking in 5% BSA/TBS for 1 h at RT and staining with primary

antibody overnight at 4°C. After incubation with a secondary antibody for 1 h at RT and were analyzed with the ImageXpress® Micro Confocal High-Content Imaging System (Molecular Devices, USA).

DNA fiber analysis

DNA fiber assay was performed as described previously (1). Briefly, cells were incubated medium containing 25 mM of CldU (Sigma-Aldrich, C6891) for 30 min, followed by replacement with medium containing 250 mM of IdU (Sigma-Aldrich, I7125) for 30 min. Cells were harvested and suspended in a solution (200 mM Tris-HCl, 50 mM EDTA, and 0.5% SDS). After spreading on coverslip and air-dried, the coverslip was fixed with methanol/acetic acid (3:1), denaturation, and blocking, prior to staining with rat anti-BrdU antibody (which detects CldU but not IdU, OBT0030, 1:2,000, AbDSerotec) and mouse anti-BrdU antibody (which detects IdU but not CldU, 7580, 1:1,000, BD Biosciences). Images of DNA fibers were acquired using a microscope (OLYMPUS BX53). The lengths of red- and green-labeled fibers were determined by FluoView3.0 software (Olympus).

Reference

1. Chen CW, Tsao N, Huang LY, Yen Y, Liu X, Lehman C, et al. The Impact of dUTPase on Ribonucleotide Reductase-Induced Genome Instability in Cancer Cells. Cell Rep. 2016;16(5):1287-99.

Supplemental figures and figure legends

Figure S1

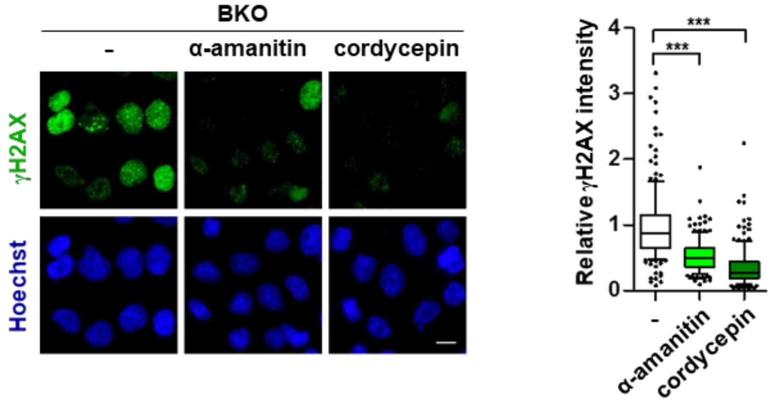

Figure S1 RNA polymerase inhibitors reduced DNA damage in BKO cells.

BKO cells were treated with RNA polymerase inhibitors α-amanitin (20 µg/ml) or cordycepin (50 µM) for 6h, followed by γH2AX IF staining with Hoechst (scale bar, 10 µm). Fluorescent intensity of γH2AX in cells (n>150) from three independent experiments was quantitated and relative intensity is expressed, ***P<0.001 by the Mann-Whitney test (*right*).

Figure S2

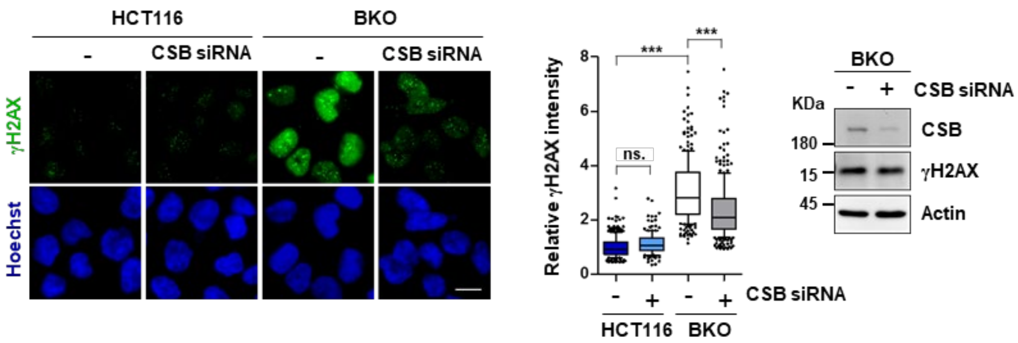

Figure S2 DNA damage in BKO cells were abolished by CSB knockdown.

HCT116 and BKO cells were transfected with 100 nM control or CSB siRNA using Lipofectamine® 2000. After post-transfection at 72 h, cells were fixed for IF staining using the γH2AX antibody (scale bar, 10 μm). Fluorescent intensity of γH2AX in cells (n>150) was quantitated by Image J from three independent experiments, and relative intensity is expressed, ***P< 0.001 by the Mann-Whitney test (*middle*). Western blots analysis of CSB, γH2AX and Actin (*right*).

Figure S3

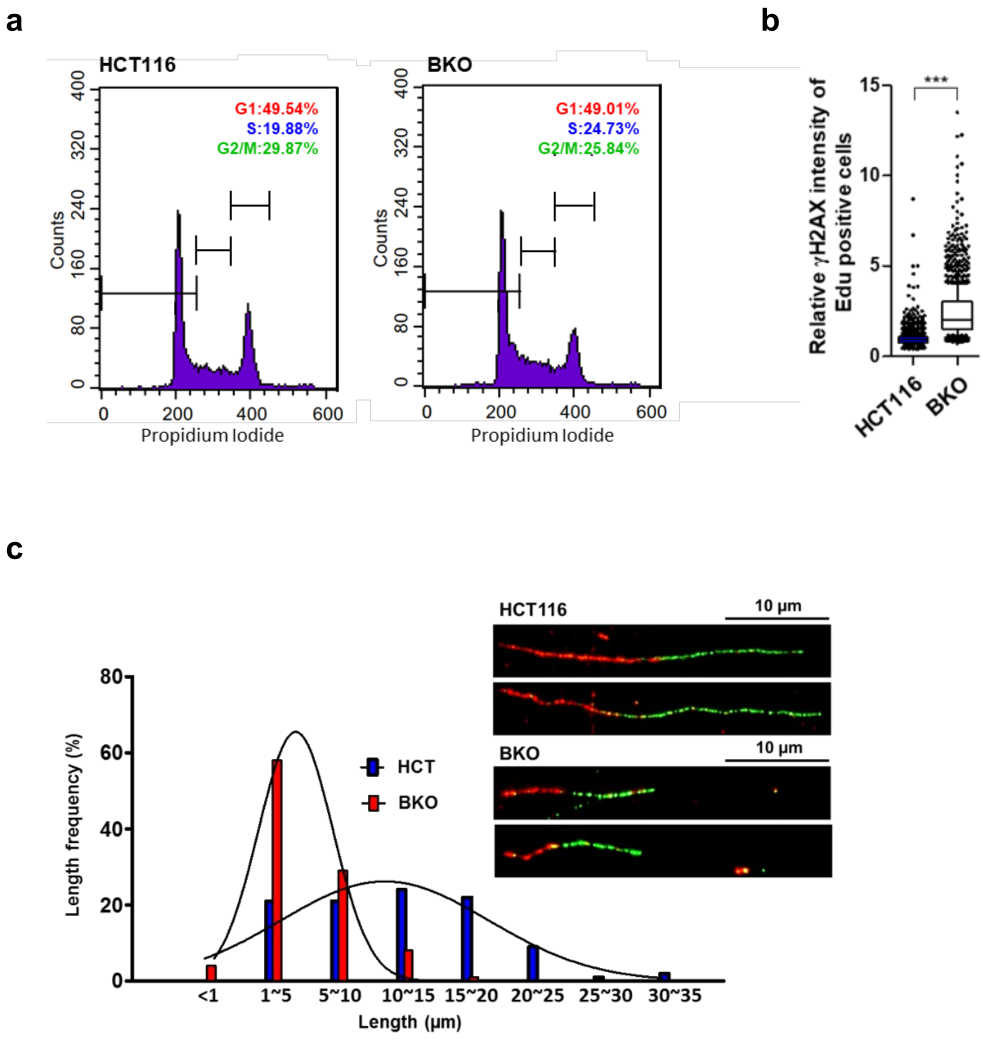

Figure S3 The replication stress in the S phase is associated with DNA damage in BKO cells.

(a) Flow cytometry profiles of DNA content of HCT116 and BKO cells. Fraction in G1 (red), S (blue), and G2/M (green) phases is indicated as % of cell population. (b) HCT116 and BKO cells were pulse-labeled with 10 μM EdU for 30 min to analyze the S phase of cells. After labeling, cells were fixed and carried out using the Click-iT™ EdU Alexa Fluor™ 488 dye Imaging Kit

according to manufacturer's protocol for image analysis. Fluorescent intensity of γH2AX in Edu-labeling cells (n>1500) was quantitated by a High-Content image analysis and relative intensity is expressed, ***P< 0.001 by the Mann-Whitney test. (c) HCT116 and BKO cells for analysis of DNA replication elongation. Cells were sequentially labeled with CldU (red) and IdU (green) for DNA spreading followed by BrdU antibody staining for DNA fiber analysis. Representative DNA fiber images are given. The length of the IdU tract connected to CIdU labeled fiber was measured (n>100).

Figure S4

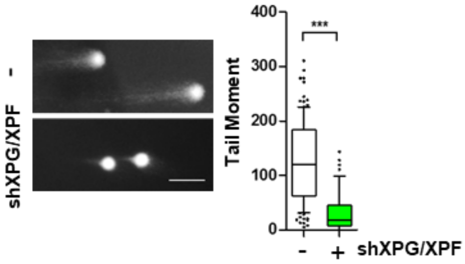

Figure S4 Comet tail in ICF cells were reduced by XPG/XPF knockdown.

ICF cells with or without XPG/XPF knockdown for comet tail moments analysis. Cells (n=300) were measured and analyzed by CometScore (***P<0.001 by the Mann-Whitney test).

Figure S5

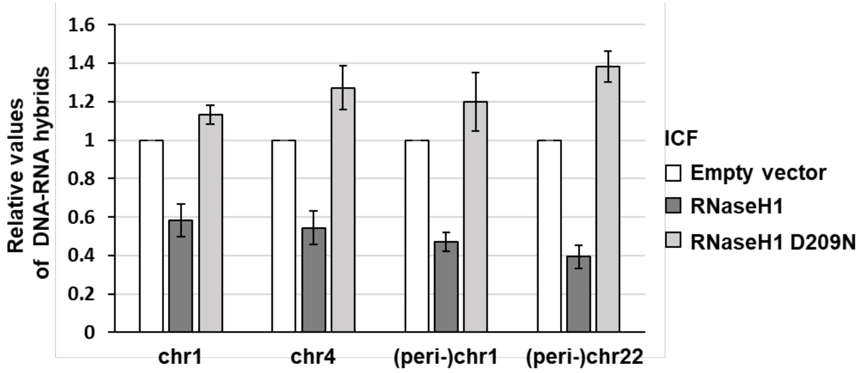

Figure S5 The DRIP assay for evaluating the level of DNA-RNA hybrid by ectopic expression of WT and catalytic-dead RNase H1 in ICF cells.

ICF LCLs were infected with retrovirus of empty vector, HA-RNaseH1-WT and HA-RNaseH1-D209N for DRIP-qPCR analysis at (peri-)centromere sequences of chromosomes as indicated. Value of DRIP-qPCR was normalized to IgG. Data are expressed relative to empty vector cells from two independent experiments.
