# Supplementary material for: DNMT3b protects centromere integrity by restricting R-loop-mediated DNA damage
Source: Cell Death Dis. 2022 Jun 11;13(6):546. doi: 10.1038/s41419-022-04989-1 (PMC9187704; doi:10.1038/s41419-022-04989-1)
Supplement: Supplementary file 2 — Supplemental material (original data-blots) [file 41419_2022_4989_MOESM2_ESM.pptx]

## Slide 1
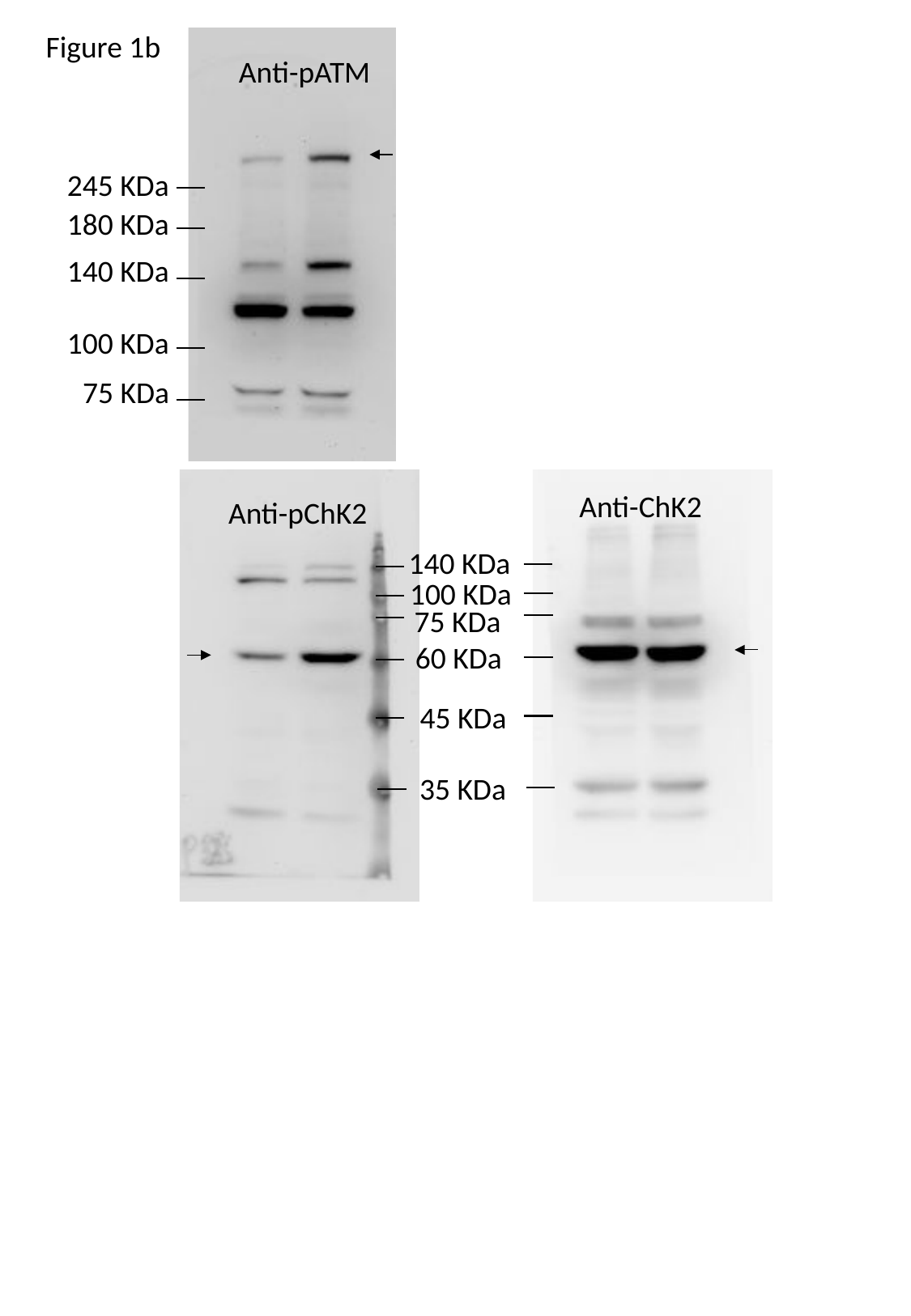

Figure 1b
Anti-pATM
245 KDa
180 KDa
140 KDa
100 KDa
75 KDa
Anti-ChK2
Anti-pChK2
140 KDa
100 KDa
75 KDa
60 KDa
45 KDa
35 KDa

## Slide 2
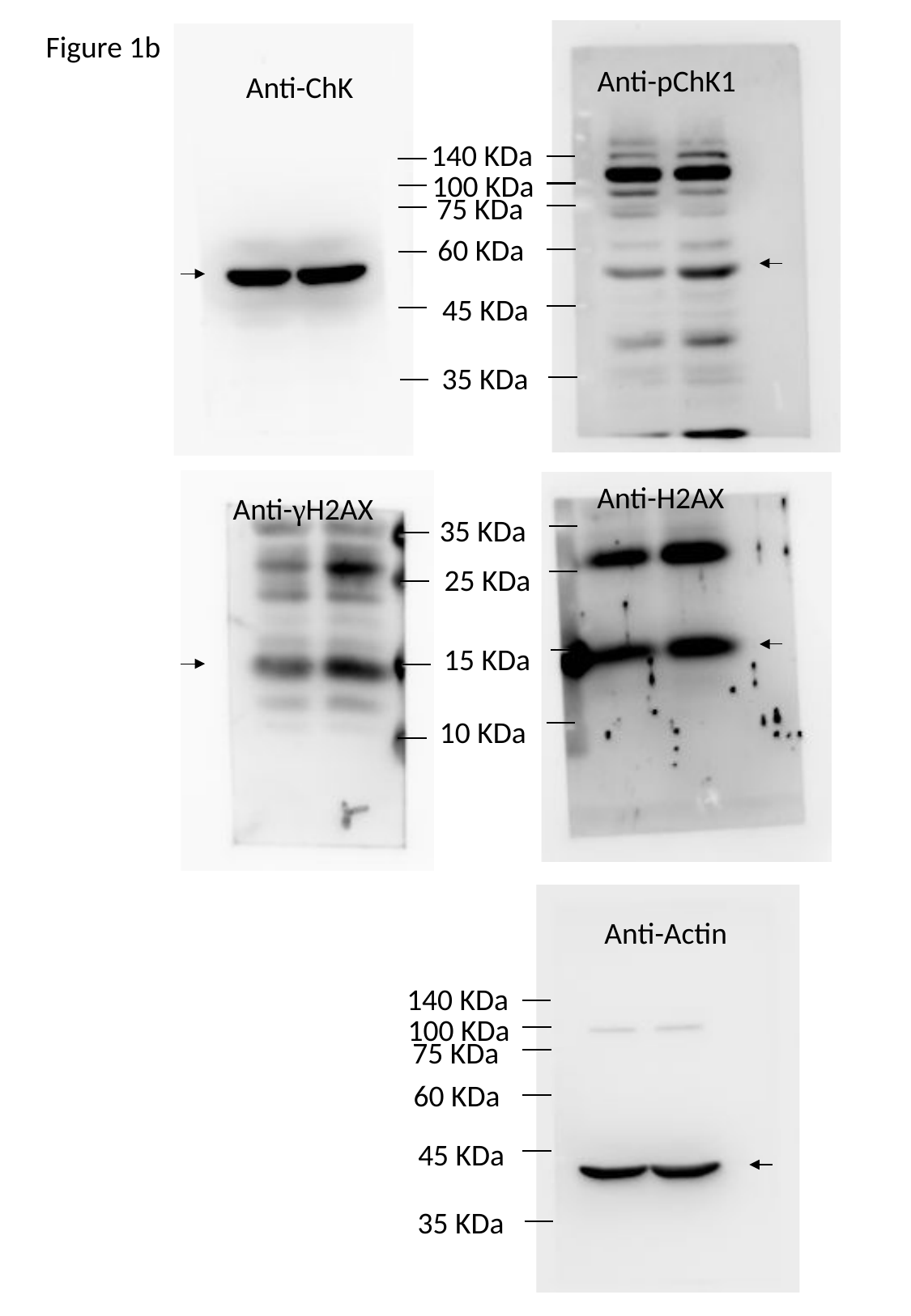

Figure 1b
Anti-pChK1
Anti-ChK
140 KDa
100 KDa
75 KDa
60 KDa
45 KDa
35 KDa
Anti-H2AX
Anti-γH2AX
35 KDa
25 KDa
15 KDa
10 KDa
Anti-Actin
140 KDa
100 KDa
75 KDa
60 KDa
45 KDa
35 KDa

## Slide 3
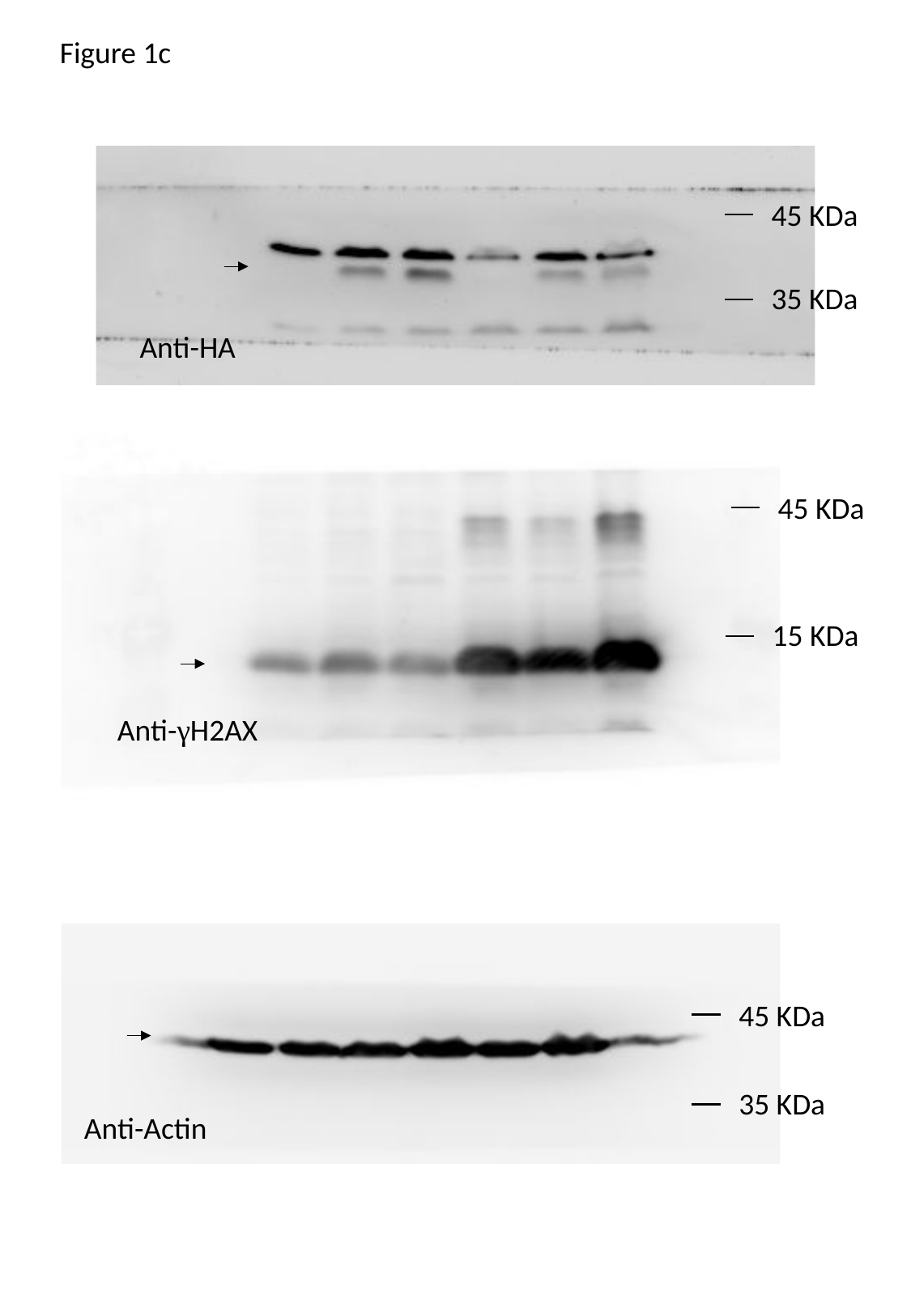

Figure 1c
45 KDa
35 KDa
Anti-HA
45 KDa
15 KDa
Anti-γH2AX
45 KDa
35 KDa
Anti-Actin

## Slide 4
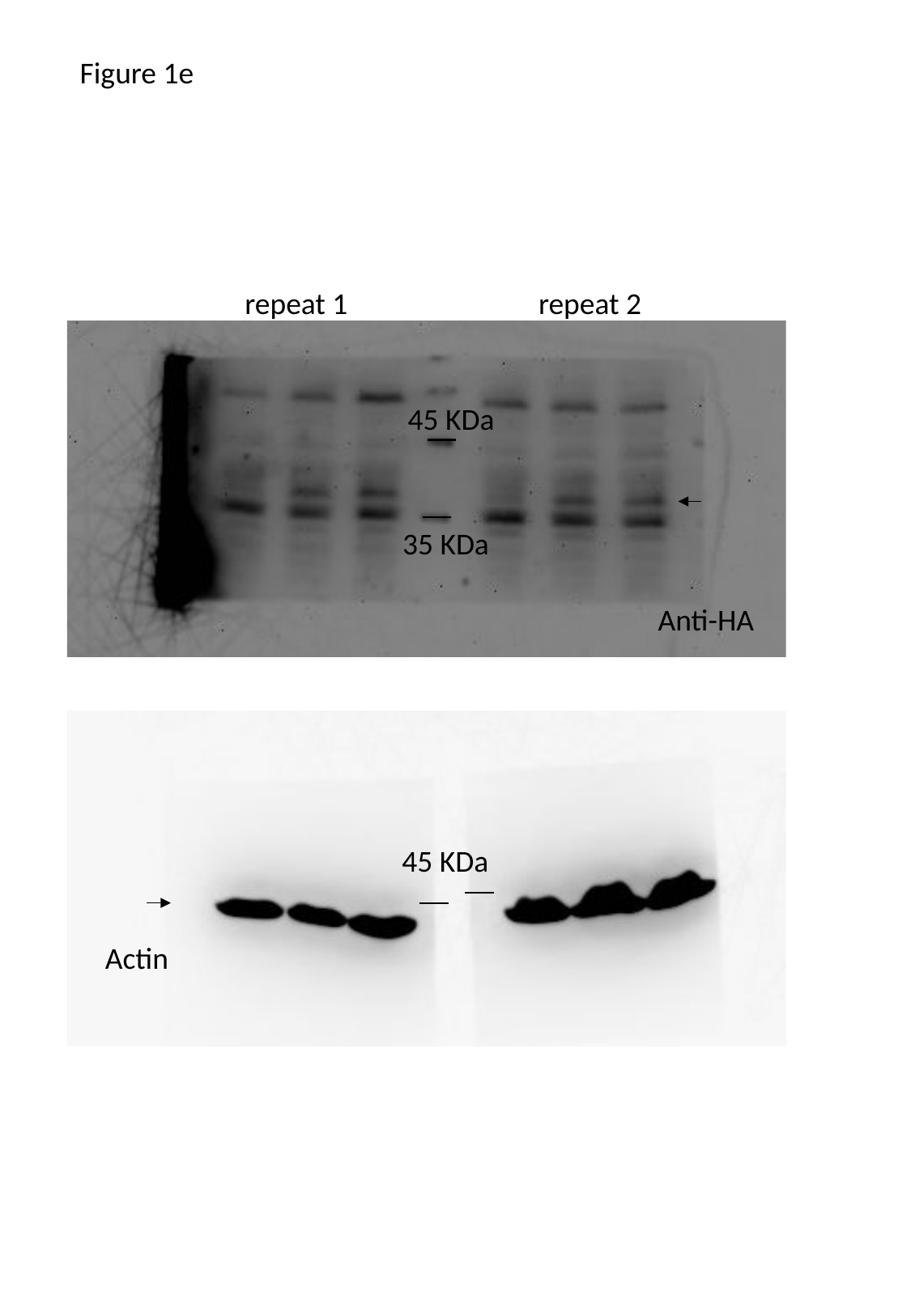

Figure 1e
repeat 1
repeat 2
45 KDa
35 KDa
Anti-HA
45 KDa
Actin

## Slide 5
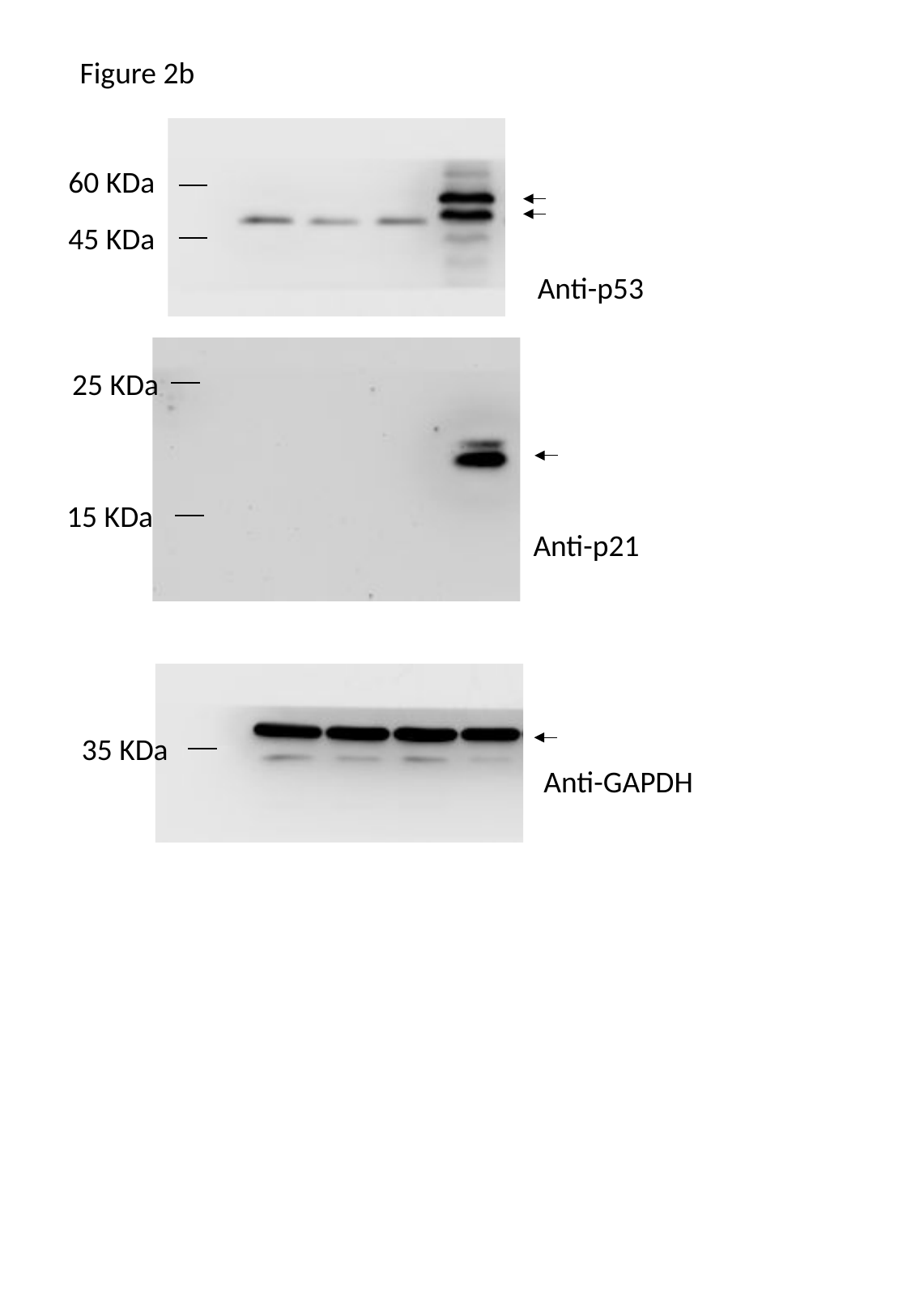

Figure 2b
60 KDa
45 KDa
Anti-p53
25 KDa
15 KDa
Anti-p21
35 KDa
Anti-GAPDH

## Slide 6
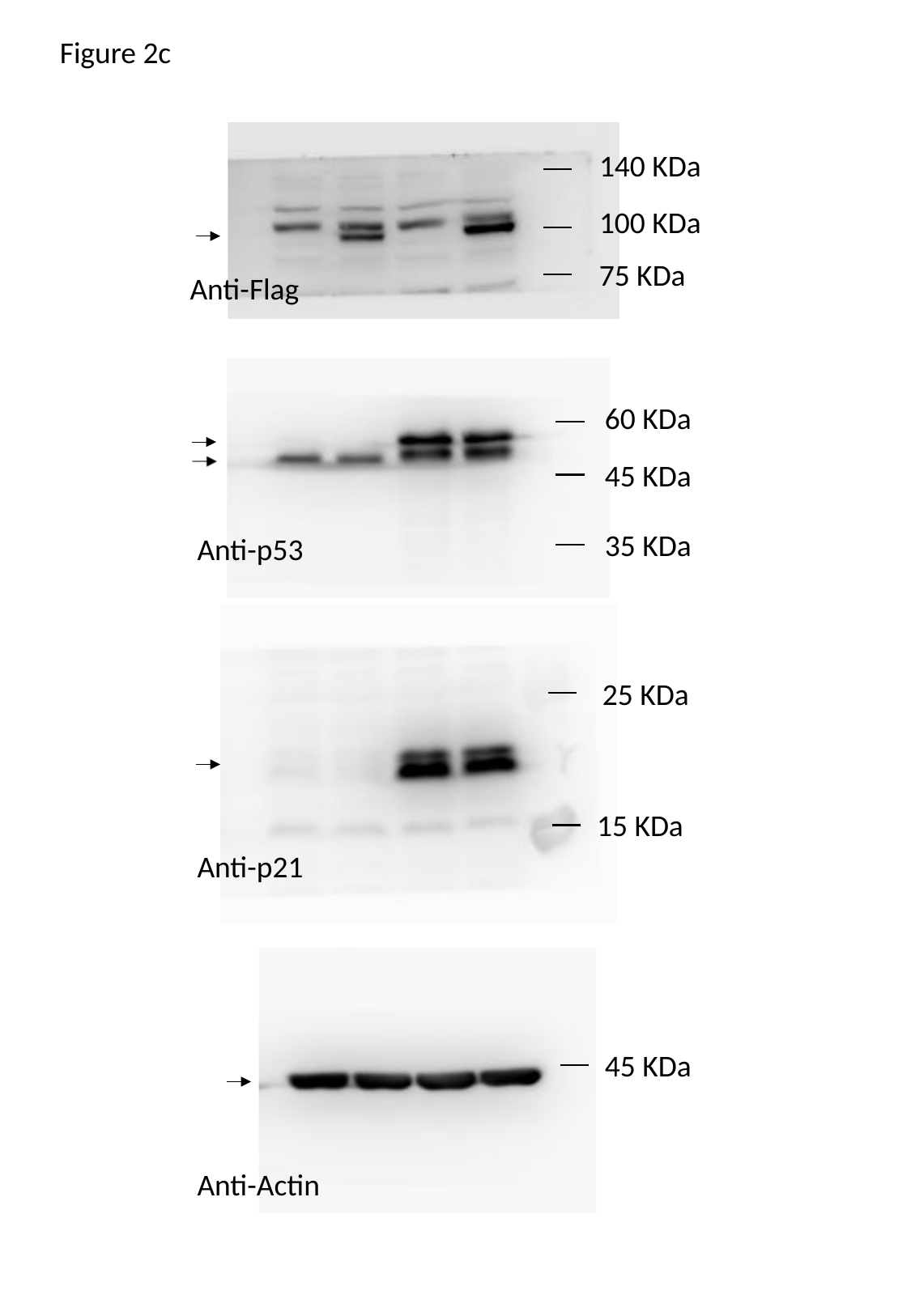

Figure 2c
140 KDa
100 KDa
75 KDa
Anti-Flag
60 KDa
45 KDa
35 KDa
Anti-p53
25 KDa
15 KDa
Anti-p21
45 KDa
Anti-Actin

## Slide 7
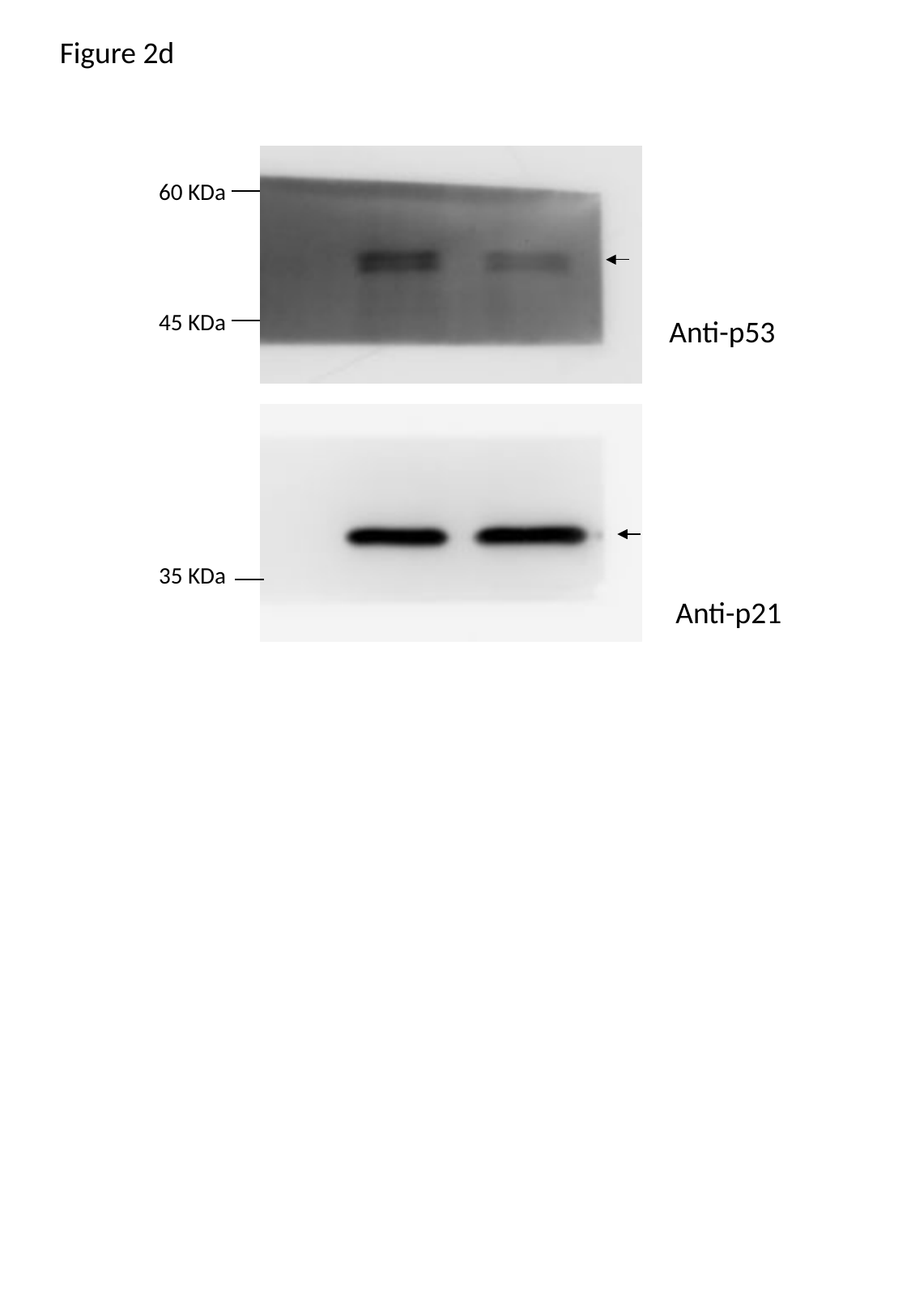

Figure 2d
60 KDa
45 KDa
Anti-p53
35 KDa
Anti-p21

## Slide 8
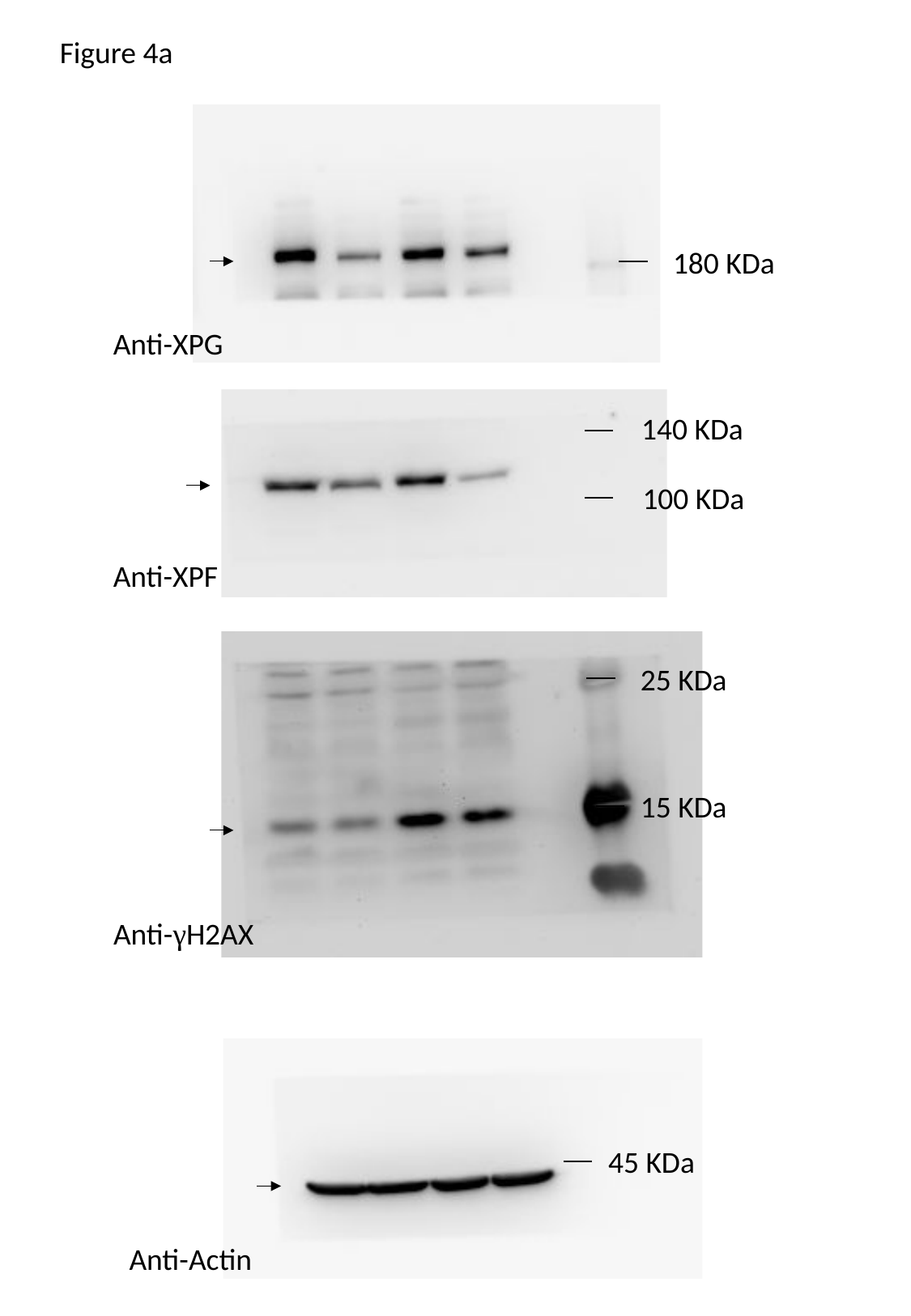

Figure 4a
180 KDa
Anti-XPG
140 KDa
100 KDa
Anti-XPF
25 KDa
15 KDa
Anti-γH2AX
45 KDa
Anti-Actin

## Slide 9
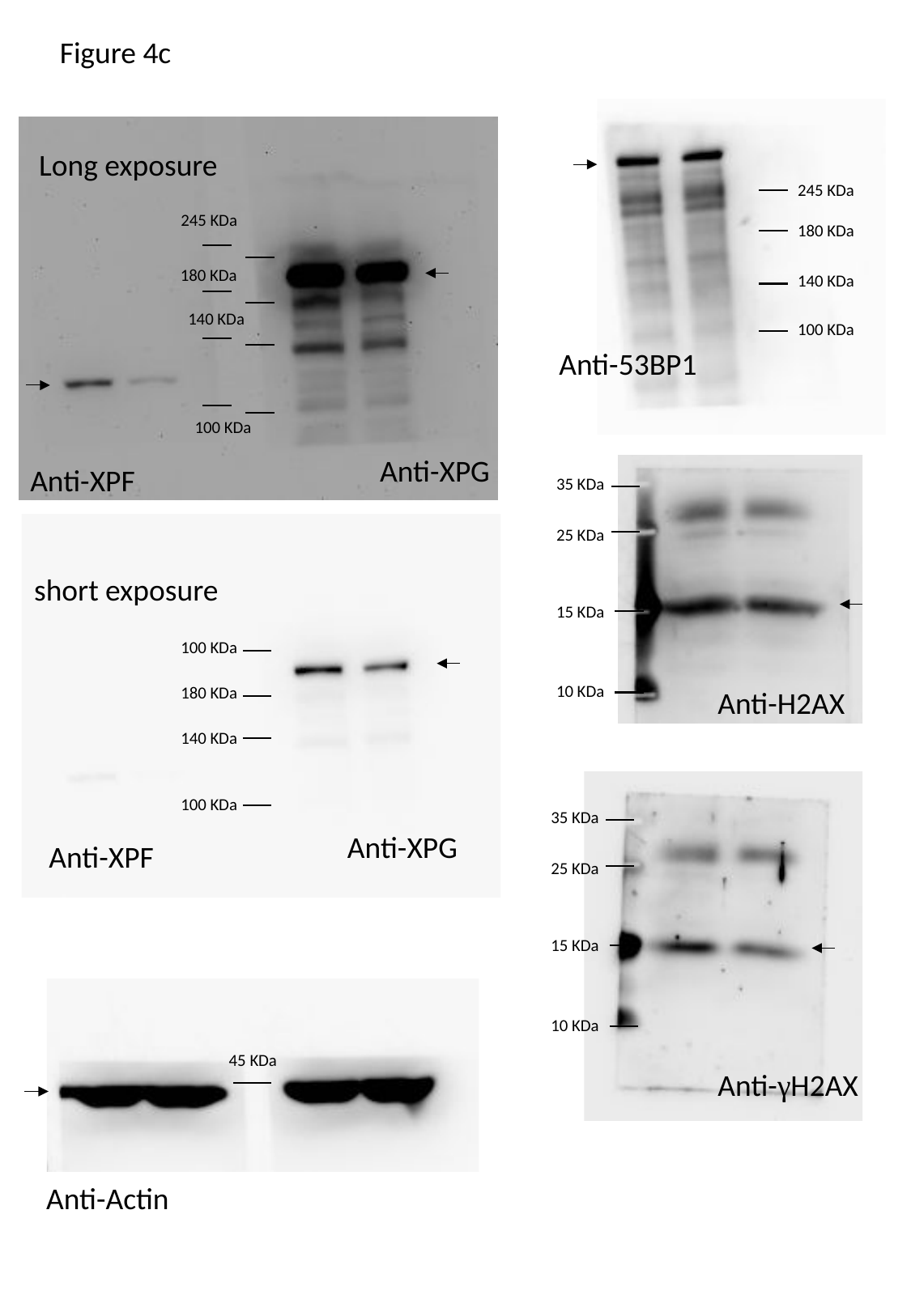

Figure 4c
Long exposure
245 KDa
245 KDa
180 KDa
180 KDa
140 KDa
140 KDa
100 KDa
Anti-53BP1
100 KDa
Anti-XPG
Anti-XPF
35 KDa
25 KDa
short exposure
15 KDa
100 KDa
10 KDa
180 KDa
Anti-H2AX
140 KDa
100 KDa
35 KDa
Anti-XPG
Anti-XPF
25 KDa
15 KDa
10 KDa
45 KDa
Anti-γH2AX
Anti-Actin

## Slide 10
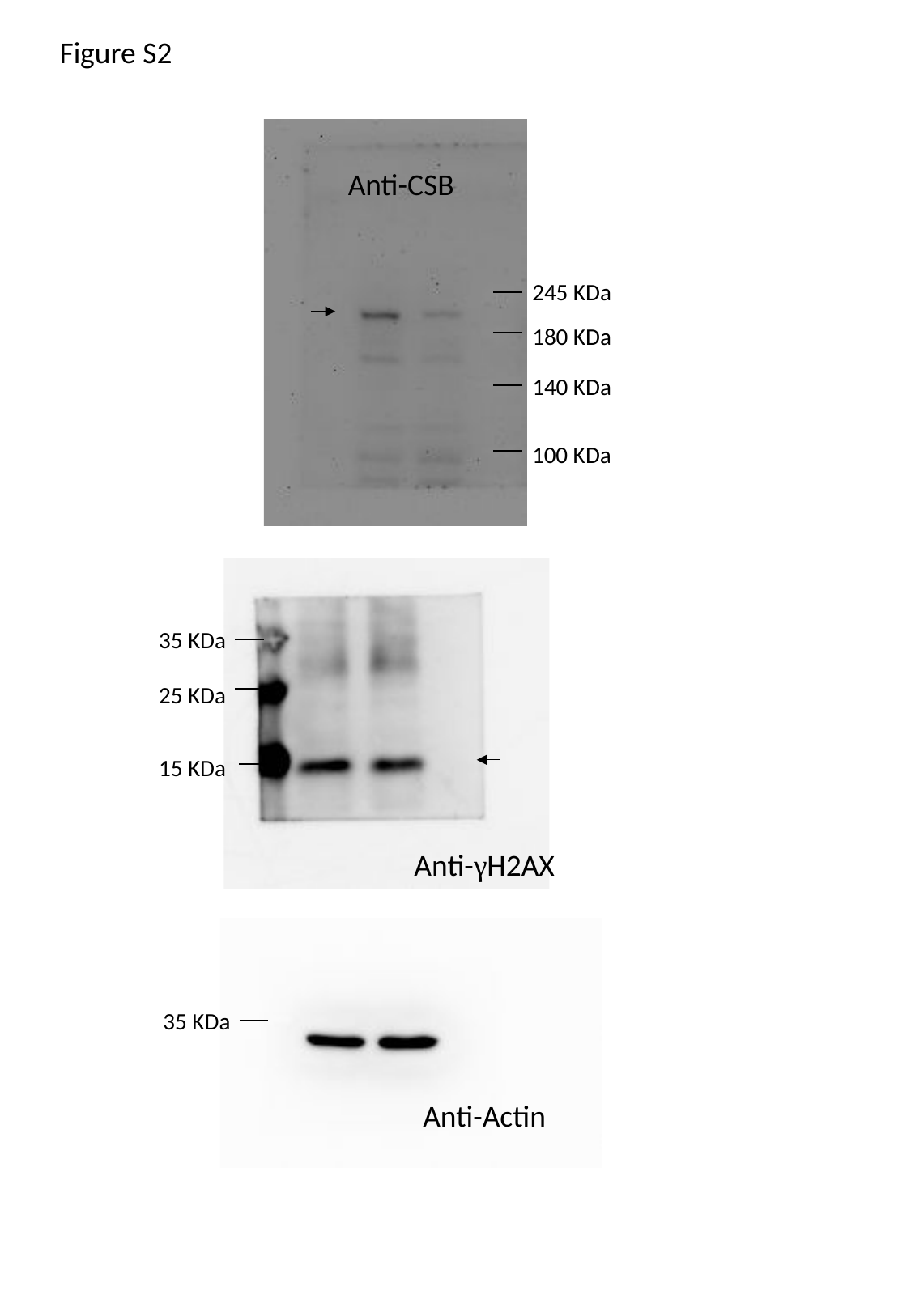

Figure S2
Anti-CSB
245 KDa
180 KDa
140 KDa
100 KDa
35 KDa
25 KDa
15 KDa
Anti-γH2AX
35 KDa
Anti-Actin
