# Supplementary figures and images for: DNMT3b protects centromere integrity by restricting R-loop-mediated DNA damage
Source: Cell Death Dis. 2022 Jun 11;13(6):546. doi: 10.1038/s41419-022-04989-1 (PMC9187704; doi:10.1038/s41419-022-04989-1)

## Slide 1
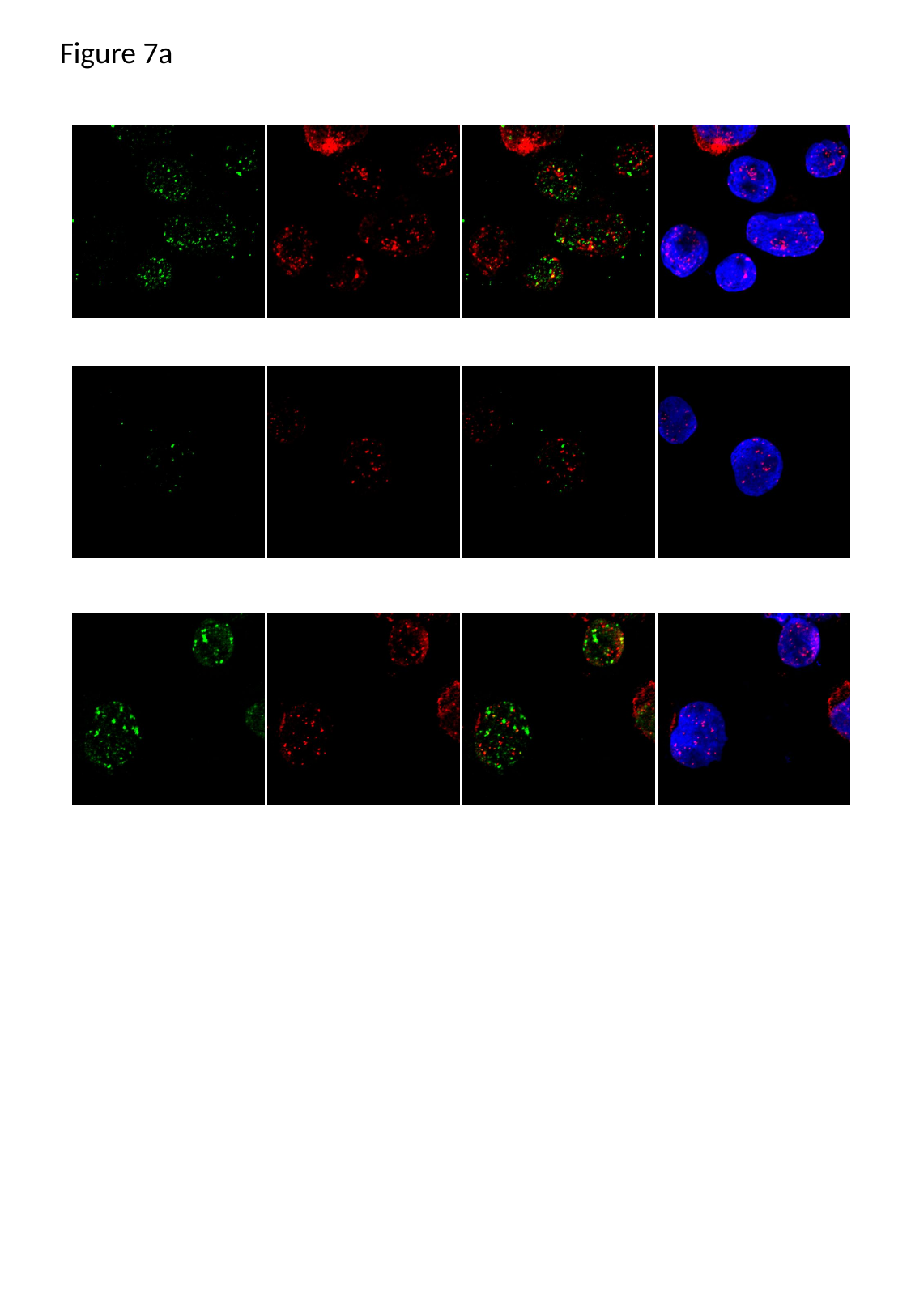

Figure 7a

## Slide 2
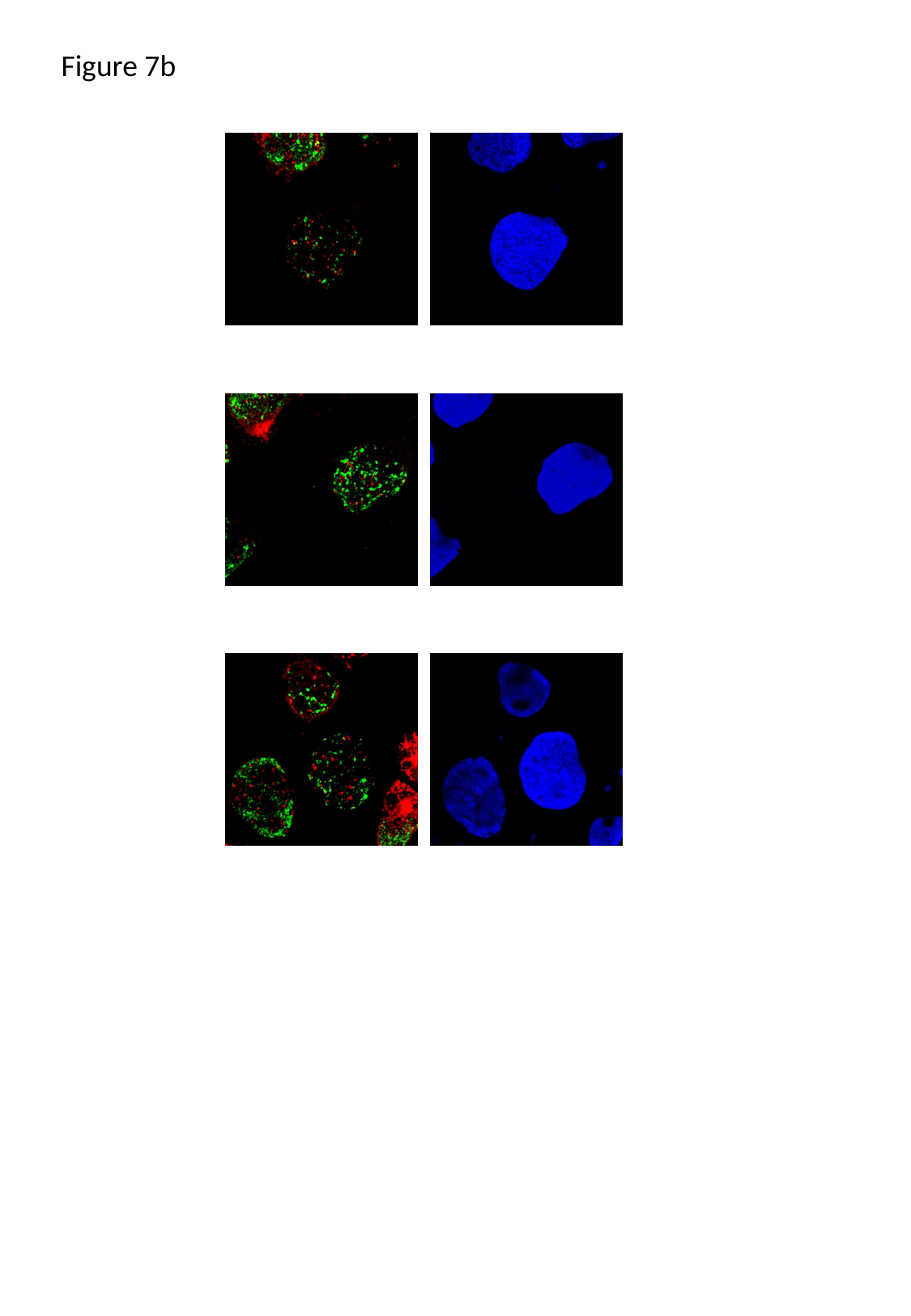

Figure 7b

Supplement: Supplementary file 4 — Original Data File [file 41419_2022_4989_MOESM4_ESM.pptx]
